# Analysis of bacterial transcriptome and epitranscriptome using nanopore direct RNA sequencing

**DOI:** 10.1093/nar/gkae601

**Published:** 2024-07-16

**Authors:** Lu Tan, Zhihao Guo, Yanwen Shao, Lianwei Ye, Miaomiao Wang, Xin Deng, Sheng Chen, Runsheng Li

**Affiliations:** Department of Infectious Diseases and Public Health, Jockey Club College of Veterinary Medicine and Life Sciences, City University of Hong Kong, Hong Kong, China; Department of Infectious Diseases and Public Health, Jockey Club College of Veterinary Medicine and Life Sciences, City University of Hong Kong, Hong Kong, China; Department of Infectious Diseases and Public Health, Jockey Club College of Veterinary Medicine and Life Sciences, City University of Hong Kong, Hong Kong, China; Department of Infectious Diseases and Public Health, Jockey Club College of Veterinary Medicine and Life Sciences, City University of Hong Kong, Hong Kong, China; Department of Infectious Diseases and Public Health, Jockey Club College of Veterinary Medicine and Life Sciences, City University of Hong Kong, Hong Kong, China; Department of Biomedical Sciences, Jockey Club College of Veterinary Medicine and Life Sciences, City University of Hong Kong, Hong Kong, China; Tung Biomedical Sciences Centre, City University of Hong Kong, Hong Kong, China; State Key Lab of Chemical Biology and Drug Discovery and Department of Food Science and Nutrition, The Hong Kong Polytechnic University, Hong Kong, China; Department of Precision Diagnostic and Therapeutic Technology, City University of Hong Kong Shenzhen Futian Research Institute, Shenzhen, 518057, China; Department of Infectious Diseases and Public Health, Jockey Club College of Veterinary Medicine and Life Sciences, City University of Hong Kong, Hong Kong, China; Tung Biomedical Sciences Centre, City University of Hong Kong, Hong Kong, China

## Abstract

Bacterial gene expression is a complex process involving extensive regulatory mechanisms. Along with growing interests in this field, Nanopore Direct RNA Sequencing (DRS) provides a promising platform for rapid and comprehensive characterization of bacterial RNA biology. However, the DRS of bacterial RNA is currently deficient in the yield of mRNA-mapping reads and has yet to be exploited for transcriptome-wide RNA modification mapping. Here, we showed that pre-processing of bacterial total RNA (size selection followed by ribosomal RNA depletion and polyadenylation) guaranteed high throughputs of sequencing data and considerably increased the amount of mRNA reads. This way, complex transcriptome architectures were reconstructed for *Escherichia coli* and *Staphylococcus aureus* and extended the boundaries of 225 known *E. coli* operons and 89 defined *S. aureus* operons. Utilizing unmodified *in vitro*-transcribed (IVT) RNA libraries as a negative control, several Nanopore-based computational tools globally detected putative modification sites in the *E. coli* and *S. aureus* transcriptomes. Combined with Next-Generation Sequencing-based N6-methyladenosine (m6A) detection methods, 75 high-confidence m6A candidates were identified in the *E. coli* protein-coding transcripts, while none were detected in *S. aureus*. Altogether, we demonstrated the potential of Nanopore DRS in systematic and convenient transcriptome and epitranscriptome analysis.

## Graphical Abstract

**Graphical Abstract:**
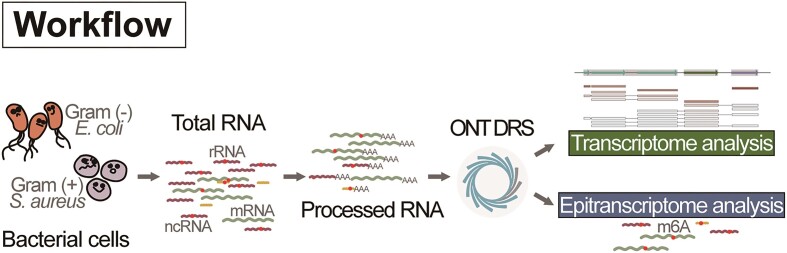

## Introduction

All steps in gene expression, including transcription, RNA processing, translation, and protein turnover, determine a cell's fate and are dedicatedly regulated. The classic view regarded bacterial gene expression as a relatively simple process. However, thanks to technological advances, especially the emergence of RNA sequencing (RNA-seq), mounting evidence has demonstrated the complexity of this process (1). In addition to providing a dynamic view of gene expression (2), RNA-seq facilitates the annotation of novel putative genes and allows better definitions of operon structures (3). Modifications to conventional RNA-seq methods further enable the high-precision analysis of RNA ends, providing insight into transcription start sites and *cis-*regulatory RNA elements (4–6). A recent study utilized a combination of these different RNA-seq approaches to investigate the transcriptome of *Borrelia burgdorferi*, the etiological agent of Lyme disease (7). It identified complex gene arrangements and uncovered abundant potential RNA regulators in this bacterial pathogen, exemplifying the important application of RNA-seq methods. However, these short read-based strategies assign termini after reconstructing full transcripts, rendering the process intricate and failing to delineate the phasing between transcript starts and ends. The development of long-read sequencing technologies, such as PacBio and Nanopore sequencing, can directly define 5′ and 3′ RNA ends after sequencing full-length transcripts. A methodology named SMRT-Cappable-seq combines the isolation of primary transcripts and PacBio sequencing, advancing the understanding of complex operon variants in *Escherichia coli* (8).

Long-read sequencing, in particular Nanopore Direct RNA Sequencing (DRS), also revolutionized the detection of RNA modifications (9). It records the ionic current changes as RNA molecules are pulled through the nanopores embedded in a membrane. Since chemical modifications can alter RNA translocation in nanopores, the modified nucleotides are directly identified in a native context (10). Before this technique, a variety of RNA-seq-based approaches were developed to map different RNA modifications (11). N6-methyladenosine (m6A), the most prevalent and conserved internal modification in eukaryotic RNAs (12), has been extensively investigated. Antibody-dependent methods, such as methylated RNA immunoprecipitation sequencing (MeRIP-Seq) (13), and orthologous antibody-independent strategies, including m6A-sensitive RNA-Endoribonuclease-Facilitated sequencing (m6A-REF-seq) (14), were designed explicitly for transcriptome-wide mapping of eukaryotic m6A. However, these methods were rarely applied to bacteria, and the bacterial epitranscriptomes remain elusive. A pioneering study demonstrated the presence of m6A in the transcriptomes of a wide range of bacterium species utilizing mass spectrometry methods (15). By applying a modified MeRIP method, the same study depicted m6A distribution on *E. coli* and *Pseudomonas aeruginosa* mRNAs in a resolution of around 23 nt. It also revealed that many m6A-modified genes are related to respiration, amino acid metabolism, stress response, and small RNAs, indicating the potential roles of m6A in these pathways. Nevertheless, precise single-nucleotide position information is lacking. Furthermore, other RNA modifications also exist in bacterial mRNAs except for m6A (16), but little is known about their locations and functions.

Taken together, bacterial gene expression is a dedicatedly regulated process and warrants more in-depth understanding. In this regard, approaches that can rapidly and comprehensively analyze the RNA biology of a bacterium of interest are vital. Applying Nanopore DRS to bacterial RNA promises to unveil the complex features of bacterial transcriptomes and epitranscriptomes simultaneously. Nonetheless, previous Nanopore research in the bacteria context focused on obtaining antibiotic resistance gene information in *Klebsiella pneumoniae* (17) or investigating the composition of transfer RNAs (tRNAs) and antisense RNAs in *P. aeruginosa* (18), lacking a systematic analysis. Despite an attempt to decode the *E. coli* transcriptome utilizing Nanopore sequencing, DRS resulted in limited mRNA reads and poorly identified transcriptomic features (19). Regarding RNA modifications, one study used Nanopore DRS to monitor stress-dependent ribosomal RNA (rRNA) modification changes in *E. coli* (20). Transcriptome-wide mapping of RNA modifications based on Nanopore DRS data has yet to be reported.

Here, Nanopore DRS was applied to representative gram-negative bacterium *E. coli* and gram-positive pathogen *Staphylococcus aureus*. We showed that explicit pre-processing of bacterial total RNA before Nanopore DRS library preparation significantly improved sequencing and mapping quality. As a result, more transcriptomic features were captured, and quantitative estimates of gene expression levels became more reliable. By synthesizing modification-free *in vitro* transcribed (IVT) RNAs as negative controls, Nanopore-based comparative computational methods identified various potential modification sites in the bacterial transcriptomes. The incorporation of MeRIP-Seq assisted in the specific prediction of m6A positions. An m6A-modified IVT RNA sample was also prepared as a positive control for the computational analysis of m6A modifications in *E. coli*. Altogether, we enhanced the performance of DRS in transcriptome analysis and explored the feasibility of utilizing DRS for the global identification of RNA modifications in the context of bacteria.

## Materials and methods

### Cell growth and RNA preparation

*E. coli* strain K-12 and *S. aureus* strain Wichita cells were purchased from the American Type Culture Collection, VA, USA. The *E. coli* cells were grown in the Luria-Bertani media to an OD600 of 0.4–0.6. Cells were harvested by centrifugation at 4°C and resuspended in TRIzol Reagent (Invitrogen, MA, USA). Following incubation at 65°C for 10 min, total RNA was isolated according to the manufacturer's instructions. The *S. aureus* cells were grown in Tryptic Soy Broth to an OD600 of 0.4–0.6. After centrifugation, the cell pellet was treated with lysostaphin for 30 min at 37°C, followed by total RNA isolation using the TRIzol Reagent. Size selection was performed using the SPRIselect Beads (Beckman Coulter, IN, USA) to remove RNA fragments smaller than 150 bp. Briefly, total RNA was diluted to a final concentration of 200 ng/μl, and 0.8 volume of SPRIselect Beads were added, followed by incubation for 5 min at room temperature. The beads were washed twice with 85% ethanol and air-dried. Residual RNA was eluted with nuclease-free water. rRNA depletion was performed according to the manufacturer's instructions using the RiboMinus™ Transcriptome Isolation Kit, bacteria (Invitrogen), which was selected based on its high processing capability. Poly(A) tailing was conducted using the *E. coli* Poly(A) Polymerase (New England Biolabs, MA, USA). Briefly, a 20-μl reaction mixture was assembled containing 1–10 μg RNA, 2 μl Reaction Buffer, 2 μl 10mM ATP, 1 μl *E. coli* Poly(A) Polymerase, and 0.5 μl RNaseOUT™ Recombinant Ribonuclease Inhibitor (Invitrogen). The reaction mixture was incubated for 20 min at 37°C, followed by a clean-up step using the VAHTS RNA Clean Beads (Vazyme, JS, PRC) according to the manufacturer's instructions. Changes in RNA size distribution were monitored using a 4200 TapeStation System (Agilent, CA, USA).

### Generation of IVT RNA

IVT RNAs resembling the endogenous transcriptomes were prepared based on previously published protocols (21) (https://www.protocols.io/view/synthesis-of-in-vitro-transcribed-rna-from-whole-b-81wgb7r7yvpk/v1). The following oligonucleotides were purchased from the BGI Genomics, SZ, PRC: template switching oligo (TSO) T7 primer, 5′-ACTCTAATACGACTCACTATAGGGAGAGGGCrGrGrG-3′, where r indicates ribonucleotide bases; T7 extension primer, 5′-GCTCTAATACGACTCACTATAGG-3′. rRNA-depleted and polyadenylated RNAs were used as templates for IVT RNA synthesis. In total, 100 ng RNA in 4 μl nuclease-free water was first annealed with 1 μl oligo(dT)_23_VN primer (10 μM, New England Biolabs) and 1 μl dNTP (10 mM, New England Biolabs) for 5 min at 70°C with the lid temperature set at ≥85°C and then held at 4°C. The reverse transcription (RT) mix was assembled containing 2.5 μl RT Buffer, 0.5 μl TSO T7 primer (75 μM) and 1 μl Template Switching RT Enzyme Mix (New England Biolabs). After adding the RT mix to the annealed RNA, the reaction mixture was incubated for 90 min at 42°C and stopped by heating for 5 min at 85°C. The RNA template was subsequently hydrolyzed by the Thermostable RNase H (New England Biolabs) according to the manufacturer's instructions. The template-switching cDNA product was purified using the VAHTS RNA Clean Beads. The second-strand cDNA synthesis reaction mixture was assembled on ice consisting of 20 μl template-switching cDNA, 25 μl Q5 Hot Start High Fidelity Master Mix (New England Biolabs), 3.75 μl T7 extension primer (50 μM), and 1.25 μl nuclease-free water. Following the initial denaturation at 95°C for 1 min, the reaction mixture was incubated at 65°C for 10 min. The resulting double-strand DNA (dsDNA) was purified using the VAHTS DNA Clean Beads (Vazyme). The volume of the beads added was three times the reaction mixture. The *in vitro* transcription step was performed using the MEGAscript Kit (Ambion, MA, USA) at 37°C for 2–4 h. For modification-free IVT RNA, the reaction mixture was composed of 2 μl each of NTPs, 2 μl Reaction Buffer, 100 ng dsDNA template in 8 μl nuclease-free water, and 2 μl Enzyme Mix. The m6A-modified IVT RNA was synthesized by replacing ATP with an equal amount of N6-Methyladenosine-5′-Triphosphate (TriLink, CA, USA). The dsDNA template was removed by treatment with TURBO DNase at 37°C for 15 min. The final IVT RNA product was purified using the VAHTS RNA Clean Beads. Its concentration was measured with NanoDrop™ One Microvolume UV-Vis Spectrophotometer (Thermo Fisher, MA, USA).

### Nanopore DRS and data processing

RNA samples, including total RNA, rRNA-depleted RNA, size-selected and rRNA-depleted RNA, modification-free IVT RNA, and m6A-modified IVT RNA, were subjected to poly(A) tailing before DRS library preparation. The libraries were constructed following the manufacturer's instructions using the SQK-RNA002 Kit (ONT, Oxford, UK). The optional RT step was performed. Sequencing was conducted on the MinION platform using the R9.4.1 Flow Cell (ONT). The resulting FAST5 files were basecalled using the Guppy workflow (v5.0.16) with the configuration of rna_R9.4.1_70bps_hac.cfg or using the Dorado workflow (v0.3.4) with the configuration of rna002_70bps_hac@v3. The basecalling results were stored as FASTQ files and were statistically analyzed with SeqKit v2.3.0 (22). Raw read features, including read length and Q score, were extracted using Pomoxis v0.3.6 (https://nanoporetech.github.io/pomoxis/). Subsequently, reads were aligned to the *E. coli* genome (GenBank Accession Number: NC_000913.3) or the *S. aureus* genome (GenBank Accession Number: CP094857.1) using minimap2 v2.17 with parameter settings ‘-ax map-ont’ (23). Mapping results (SAM files) were converted into BAM files and sorted and indexed using SAMtools v1.7 (24). Read alignment information was extracted from the sorted BAM files and converted into BED files using a custom Python script based on Pysam (https://github.com/pysam-developers/pysam).

### Transcriptome analysis

First, reads reversely mapped to an annotated region were discarded. The remaining reads were further filtered based on the length (greater than 100 bp) or proportion (exceeding 50%) of overlap with the target gene sequences. This process might assign multiple alignment features to a single read, and the combination of adjacent genes within one read was considered a unique transcript type. The number of different transcript types a gene appeared in was defined as transcriptional context.

Gene expression correlations between Nanopore DRS and NGS RNA-seq datasets were subsequently calculated based on the mapping results. For DRS data, reads were filtered, followed by counting the read numbers aligned to individual genes. Two RNA-seq datasets were referenced from (25,26) and mapped to the *E. coli* genome using BWA MEM with the default parameters (27). Genes other than Protein-coding ones were removed from the counting results. Undetected genes were also excluded from the analysis. Afterward, transcripts per million (TPM) were computed for each gene in different samples using the formula, followed by pairwise calculation of Spearman's rank correlation coefficients.


\begin{equation*}TP{M_i} = \frac{{{q_i}/{l_i}}}{{\mathop \sum \nolimits_j \left( {{q_j}/{l_j}} \right)\;}}*{10^6}\end{equation*}

where *q_i_* denotes reads mapped to a gene, *l_i_* is the gene length and $\mathop \sum \limits_j ( {{q_j}/{l_j}} )$ corresponds to the sum of mapped reads to individual genes normalized by corresponding gene lengths.

### Epitranscriptome analysis using computational tools

Several single-mode computational tools have been designed to exclusively detect eukaryotic m6A modifications in the DRACH/RRACH motifs (28,29). However, these methods do not apply to bacterial epitranscriptomes since the motif preference for bacterial m6A modifications remains unclear. Therefore, only comparative methods were chosen in the present study to identify potential modification sites in the *E. coli* and *S. aureus* transcriptomes. Four error rate-based tools, including Differr (v0.2) (30), DRUMMER (v1.0) (31), ELIGOS2(v2.1.0) (32), and EpiNano_Error (v1.2) (33), and three signal level-based approaches, including Nanocompore (v1.0.4) (34), Tombo_com (1.5.1) (35), and xPore (v2.1) (36), were applied. Comparisons were made between native RNAs and modification-free IVT RNAs or between m6A-modified and modification-free IVT RNAs. Default parameter settings and cutoff thresholds for individual methods were implemented accordingly. Sites outside annotated gene bodies were excluded from analyses. Gene ontology (GO) analysis was conducted with the PANTHER knowledgebase (https://pantherdb.org/tools/compareToRefList.jsp). Motif analysis was performed using Homer (http://homer.ucsd.edu/homer/ngs/peakMotifs.html) with parameter settings ‘-size -5,5 -len 4,5,6,7 -norevopp -rna -bg < peak/BED file> -noweight -nlen 0’ (37). And nanoCEM (0.0.5.8)(https://github.com/lrslab/nanoCEM) was utilized to showcase the current feature using the f5c_ev mode with the parameter settings ‘--norm --rna --pore r9’ (38).

### Library construction and bioinformatics analysis for MeRIP-Seq

Modification-free IVT RNAs were processed in parallel as rRNA-depleted RNA samples for systematic calibration (21). RNA samples (1 μg per reaction) were sheared into approximately 100-nt fragments using the RNA Fragmentation Reagents (Ambion) at 70°C for 5.5 min, followed by ethanol precipitation. Glycogen, RNA grade (Thermo Scientific), was applied as an inert carrier to increase the recovery of RNA fragments. A small amount of purified RNA fragments were aliquoted as inputs. RNA immunoprecipitation (RIP) reaction was performed using the Magna RIP™ RNA-Binding Protein Immunoprecipitation Kit (Sigma-Aldrich, MA, USA). For each reaction, 50 μl protein A/G magnetic beads were washed twice with 150 μl RIP Wash Buffer per time and resuspended in 100 μl RIP Wash Buffer. m6A antibody (10 μg per reaction, Cat. No. 202003, Synaptic Systems, DEU) were added to the beads, followed by incubation on a ThermoMixer C (Eppendorf, HH, DEU) at 1000 rpm for 30 min at room temperature. After washing three times with 500 μl RIP Wash Buffer per time, the antibody-bead complex was resuspended in 900 μl RIP Buffer (35 μl 0.5M EDTA and 5 μl RNase Inhibitor in 860 μl RIP Wash Buffer). Purified RNA fragments in 100 μl nuclease-free water were added to the antibody-bead complex and incubated at 1000 rpm overnight at 4°C. After the RNA-antibody-bead complex was washed six times with 500 μl RIP Wash Buffer per time, 100 μl Elution Buffer was added to the complex containing 150 mM NaCl, 10 mM Tris-HCl (pH 7.4), 0.1% IGEPAL CA-630, and 6.7 mM m6A 5′-monophosphate sodium salt (Sigma-Aldrich), supplemented with 7 μl RNase inhibitor. The mixture was incubated at 1000 rpm for 1 h at 4°C. The elution step was repeated twice (three times in total), and 300 μl elutes were combined and subjected to ethanol precipitation. The resulting RIP products and the input samples kept before RIP (50 ng per reaction) were used to construct NGS libraries using the VAHTS Universal V6 RNA-seq Library Prep Kit for Illumina (Vazyme) following the stranded transcriptome library protocol. The prepared NGS libraries were sent to Novogene (Kowloon, HK, CHN) for paired-end sequencing.

Raw data from MeRIP-Seq was trimmed to remove adapters using Trimmomatic v0.39 (39). Data quality control was completed with FastQC (https://www.bioinformatics.babraham.ac.uk/projects/fastqc/). Clean reads were aligned to the *E. coli* or *S. aureus* genomes using BWA MEM with the default parameters (27). The resulting SAM files were converted into BAM files using SAMtools v1.16 with the ‘-F 260’ parameter to keep high-quality mapping results (24). exomePeak2 v1.9.1 was applied for peak calling with the parameter settings ‘p_cutoff = 0.00001, log2FC_cutoff = 1, fragment_length = 150’. The m6A peaks in the modification-free IVT RNA libraries were defined as false positives and applied to calibrate m6A peaks detected in the rRNA-depleted RNA libraries (21).

## Results

### Pre-processing of total RNA significantly improved the sequencing and mapping quality of bacterial transcriptome

Unlike eukaryotic mRNAs, bacterial mRNAs lack a poly(A) tail, which is required to capture the mRNA populations and is an essential prerequisite for conducting DRS. Therefore, rRNAs, which account for more than 85% of prokaryotic cellular RNA contents, are usually depleted before library preparation to increase the proportion of sequenced mRNAs (40). Bacterial RNAs also need to be enzymatically polyadenylated to enable the ligation of sequencing adapters (18). However, according to our experiences and other publications (17), these treatments sometimes compromised sequencing and mapping qualities.

**Figure 1.**
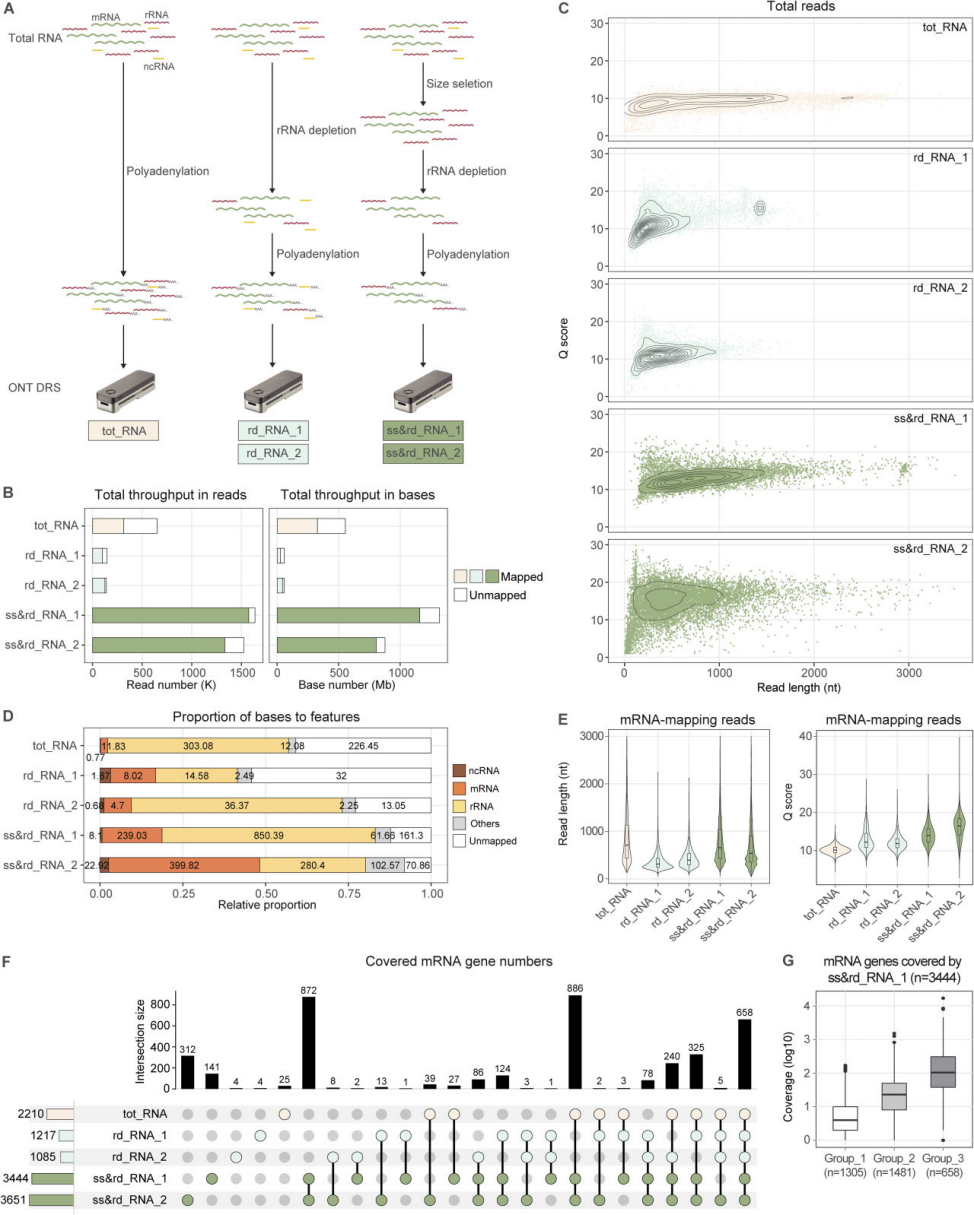
Raw read features and analysis of mapped reads based on Dorado basecalling results. (**A**) Datasets obtained with differentially processed RNA samples. *E. coli* strain K12 total RNAs were isolated from cells grown to a log phase. Before Oxford Nanopore Technologies (ONT) Direct RNA Sequencing (DRS) library preparation, total RNAs were directly polyadenylated (tot_RNA), experienced rRNA depletion followed by polyadenylation (rd_RNA), or underwent size selection prior to rRNA depletion and polyadenylation (ss&rd_RNA). mRNA, messenger RNA; rRNA, ribosomal RNA; ncRNA, non-coding RNA. (**B**) Total sequencing throughput. FAST5 files were basecalled using the Dorado model, and the resulting FASTQ files were aligned to the reference genome using minimap2. (**C**) Relationship between read length and quality of raw reads shown as a 2D density plot. Read qualities were specified by Q scores. (**D**) Proportion of bases to annotation features. Numbers embedded in the bars indicate the amount of bases in each category. (**E**) Read length and quality of mRNA-mapping reads. (**F**) UpSet plot showing the intersections of covered mRNA genes by individual datasets. Only protein-coding genes were included in the analysis (*n* = 4288). (**G**) Coverage depth of different genes in the ss&rd_RNA_1 dataset. Genes detected in the ss&rd_RNA_1 library were grouped into three categories: those only detected in ss&rd_RNA_1, those detected in ss&rd_RNA_1 and rd_RNA, and those detected in ss&rd_RNA_1, rd_RNA, and tot_RNA.

**Figure 2.**
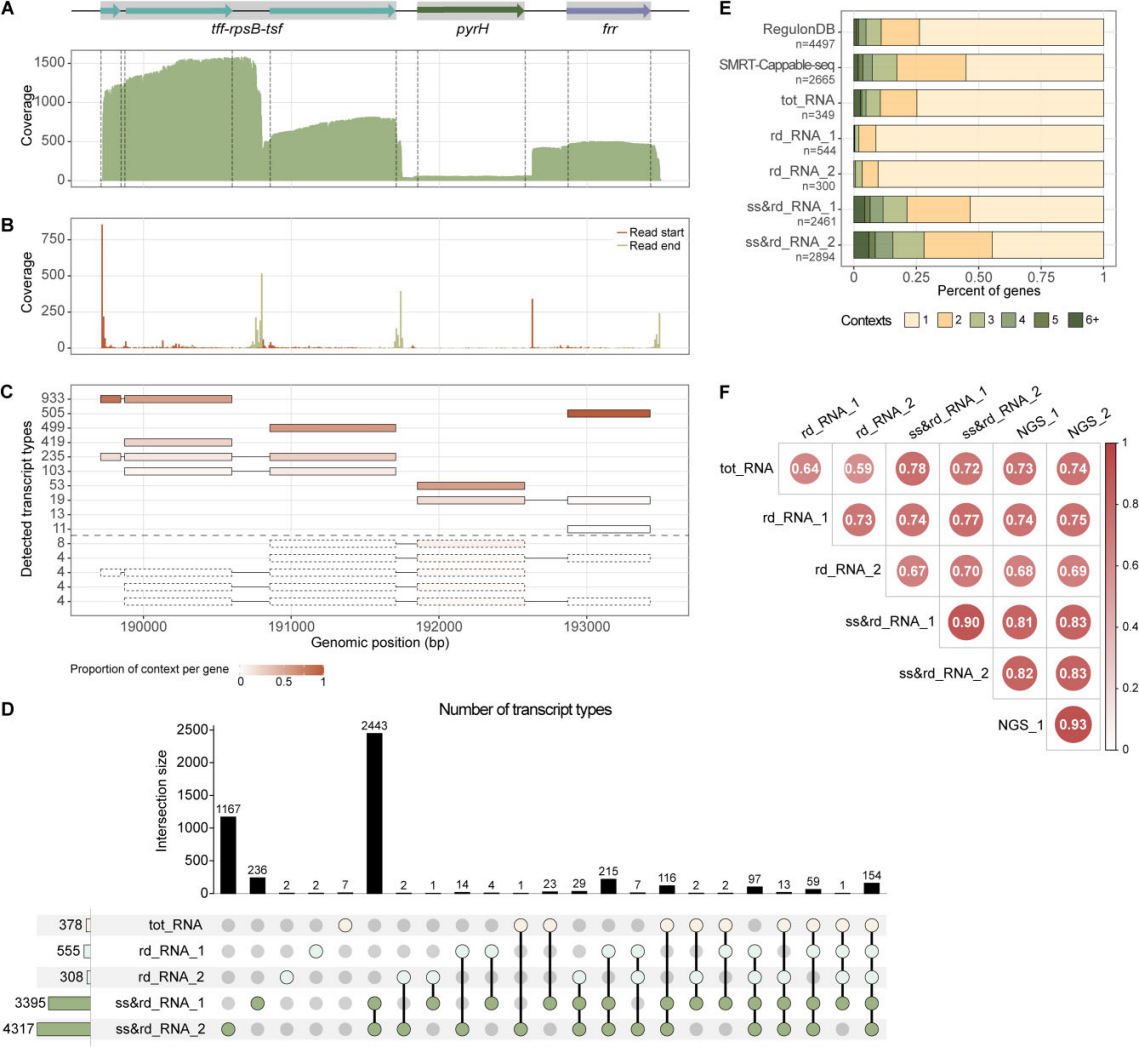
*E. coli* transcriptomic features identified by ONT DRS. (**A**) Coverage plot of reads aligned to the *tff-rpsB-tsf* operon and neighboring regions in the ss&rd_RNA dataset. Gray shallows indicate the gene contents of defined operons in RegulonDB. (**B**) Visualization of read boundaries. Bin size = 10. (**C**) Different types of transcripts identified in the *tff-rpsB-tsf* operon and neighboring regions. Transcript type is defined as a unique combination of genes covered by a read. The amount of reads assigned to each transcript type is shown on the left. The distribution of each gene in different contexts is visualized using a color code. It was calculated by dividing the number of reads assigned to a context by the total number of reads aligned to the gene. (**D**) UpSet plot showing the number and comparability of transcript types detected in different datasets. Transcript types supported by >10 reads were included in the analysis. (**E**) Percent of genes presenting in different transcriptional contexts. The number of transcriptional contexts is defined as the number of transcript types a gene is a part of. The full list of transcriptional units deposited in the RegulonDB database was downloaded and computed as a control (42). The SMRT-Cappable-seq dataset was pooled from (8). (**F**) Correlation matrix of protein-coding gene expression levels between tot_RNA, rd_RNA, ss&rd_RNA and two Next-Generation Sequencing (NGS)-based RNA Sequencing (RNA-Seq) data. Spearman's rank correlation coefficients were calculated using the transcripts per million (TPM) values. Each library has excluded the rRNA reads before TPM counting.

**Figure 3.**
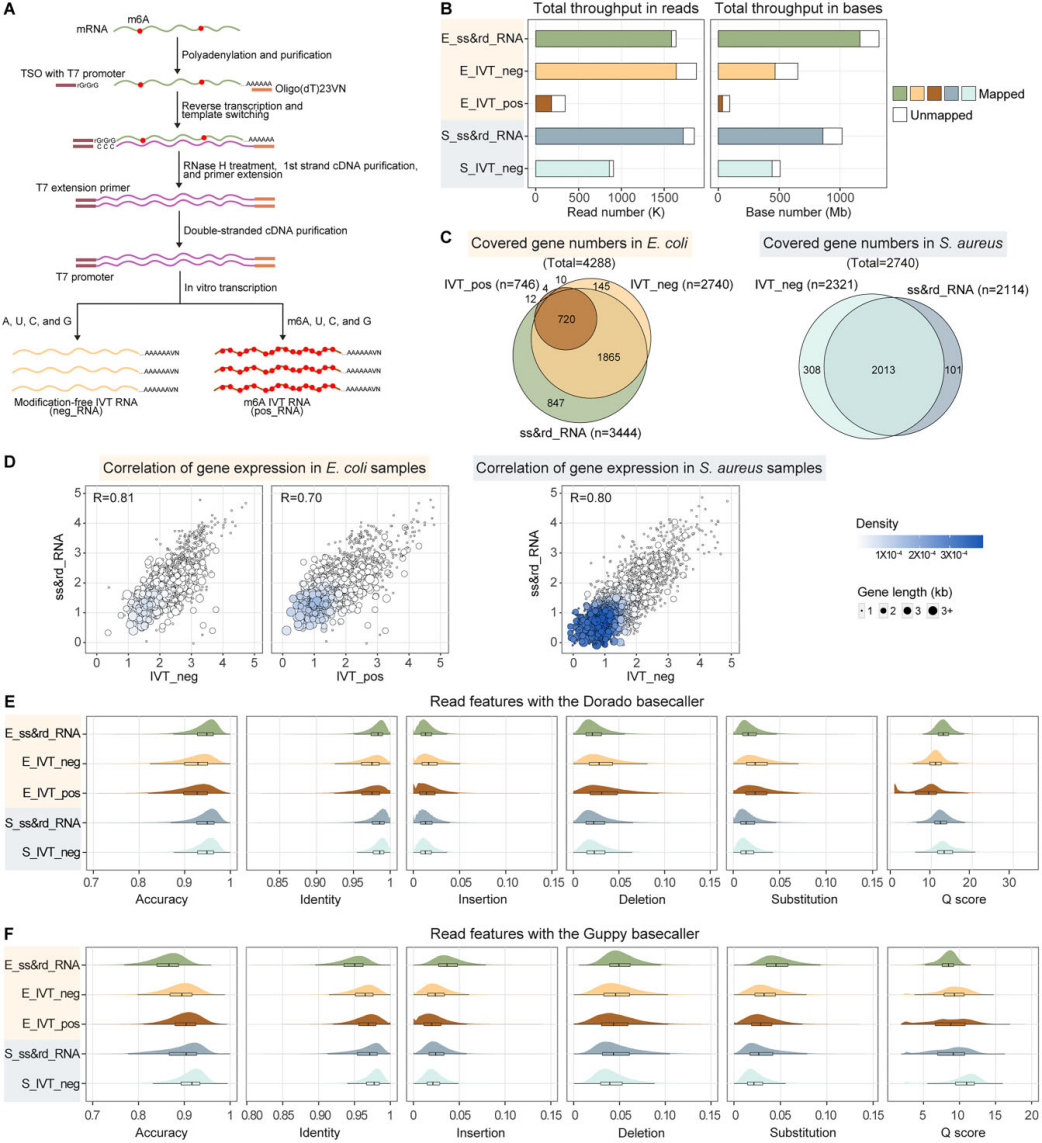
Generation of transcriptome-wide modification-free and m6A-modified IVT RNAs. (**A**) Workflow of IVT RNA synthesis. Briefly, rRNA-depleted RNAs are polyadenylated, followed by synthesizing double-stranded cDNA molecules, which serve as templates for *in vitro* transcription. Unmodified nucleotides are used for modification-free RNA synthesis. m6A-modified RNAs are synthesized by replacing unmodified A with m6A in the reaction mixture. (**B**) Total sequencing throughput of the native and IVT datasets. E_ss&rd_RNA and S_ss&rd_RNA indicate the sequencing results of *E. coli* and *S. aureus* native RNAs, respectively. E_IVT_neg and S_IVT_neg indicate the sequencing results of *E. coli* and *S. aureus* modification-free IVT RNAs, respectively. E_IVT_pos shows the sequencing result of *E. coli* m6A-modified IVT RNAs. DRS FAST5 files were basecalled using the Dorado model. The resulting FASTQ files were aligned to respective reference genomes using minimap2. (**C**) Venn diagrams showing the intersections of genes covered by the native and corresponding IVT datasets. (**D**) Correlations of protein-coding gene expression levels between the native and IVT samples. Spearman's rank correlation coefficients were calculated using TPM values. Undetected genes were excluded from the analyses. Each point represents one gene, color-coded by the density at the plot position. Gene length is indicated by the point size. (**E**) Read metrics of native and IVT reads basecalled using the Dorado basecaller. Read-level accuracy, identity, insertion, deletion, and substitution were calculated based on the mapping results, while the Q score was plotted using all reads. (**F**) Read metrics of native and IVT reads basecalled using the Guppy basecaller.

Here, total RNAs were isolated from the prokaryotic model organism *E. coli* strain K-12 grown to a log phase. Three different pre-processing procedures were applied before DRS library preparation (Figure 1A). Total RNAs were either directly polyadenylated (tot_RNA) or subjected to rRNA depletion followed by polyadenylation (rd_RNA). Another group of total RNAs underwent size selection before rRNA depletion and polyadenylation (ss&rd_RNA) to remove small-sized, highly structured RNA molecules. The depletion of rRNA and small-sized RNA (<150 nt) was confirmed by TapeStation (Supplementary Figure S1). Following DRS, the raw current signal data were basecalled using either the Guppy or Dorado software. Across all datasets, Dorado with the rna002_70bps_hac@v3 model generated more accurate basecalling results than Guppy. Therefore, read features and transcriptome analyses based on the Dorado outputs are presented in this text (Table 1 and Figures 1–3). Corresponding Guppy data is shown in Supplementary Tables S1 and S2 and Supplementary Figure S2. It is seen that samples pre-processed with different procedures generated wildly varied sequencing throughputs (Table 1 and Figure 1B). rd_RNA yielded the fewest reads and bases. The average throughput of two rd_RNA replicates was approximately 58 Mb in bases. By contrast, tot_RNA and ss&rd_RNA produced about 553 Mb and 1099 Mb data, respectively. Regarding read length and quality, ss&rd_RNA significantly outperformed tot_RNA and rd_RNA (Figure 1C). Many ss&rd_RNA reads were simultaneously equipped with long read lengths and high Q score values (Figure 1C), indicating an outstanding sequencing quality of these samples.

**Table 1.** Raw read features and mapping statistics based on Dorado basecalling outputs

	*E. coli* ^a^ tot_RNA	*E. coli* rd_RNA_1	*E. coli* rd_RNA_2	*E. coli* ss&rd_RNA_1	*E. coli* ss&rd_RNA_2	*E. coli* IVT_neg	*E. coli* IVT_pos	*S. aureus* ^b^ ss&rd_RNA	*S. aureus* IVT_neg
Read number	650 546	145 946	138 567	1 636 856	1 524 490	1 875 879	342 693	1 850 019	906 942
Base number	554 196 801	58 964 409	57 052 299	1 320 489 758	876 568 459	652 980 782	94 500 310	1 017 691 598	507 500 048
Average length	852	404	412	807	575	348.1	275.8	550	560
Median length	744	290	373	706	468	322	191	444	489
N50 length	1 254	482	473	965	782	373	297	616	635
Median Q score	9.21	11.18	10.97	12.94	15.42	11.06	9.39	12.26	13.14
Ratio of mapped reads (%)	48.60	69.19	89.92	96.81	87.45	87.42	53.74	92.99	94.75
Ratio of mapped bases (%)	59.32	46.12	77.41	88.11	91.91	71.45	36.68	84.41	96.94
Ratio of reads mapped to mRNA (%)	2.68	22.01	11.04	22.09	44.53	16.3	22.28	22.2	34.36
Ratio of bases mapped to mRNA (%)	2.16	13.89	8.42	18.37	45.61	14.31	15.63	17.38	32.73

^a^ *E. coli*, *Escherichia coli*.

^b^ *S. aureus*, *Staphylococcus aureus*.

Unfiltered Dorado-basecalled reads were aligned to the *E. coli* genome using minimap2. As a result, 48.60% of tot_RNA, 79.55% of rd_RNA and 92.13% of ss&rd_RNA reads were mapped, corresponding to 59.32%, 61.76% and 90.01% of bases, respectively. In contrast, a previous study sequenced two *E. coli* total RNA samples via the Nanopore DRS platform (19), where the mapped reads accounted for only approximately 30% and 65% of the total reads. Mapped bases were classified based on their annotation features (Figure 1D). It was seen that rRNA depletion indeed increased the proportion of sequenced mRNAs from 2.68% of tot_RNA reads to 16.52% of rd_RNA reads and 33.31% of ss&rd_RNA reads, corresponding to 2.16%, 11.15% and 31.99% of bases, respectively. Meanwhile, it is notable that rRNA depletion efficiency varied in replicate samples. In our experience, vigorous and frequent tapping of the tube during RNA and bead incubation could help improve rRNA removal efficiency when using the RiboMinus kit. Among the rRNA-depleted samples, the median length of messenger RNA (mRNA)-mapping reads in ss&rd_RNA was approximately twice that in rd_RNA (Figure 1E). mRNA-mapping reads in ss&rd_RNA also showed higher read quality than in rd_RNA (Figure 1E). Given the higher sequencing throughput and mRNA-mapping ratio (Table 1), mRNA reads in ss&rd_RNA considerably outnumbered those in rd_RNA and tot_RNA. The amount of mRNA reads in ss&rd_RNA (n = 519 914) was approximately 22-fold that in rd_RNA (*n* = 23 710) and 30-fold that in tot_RNA (*n* = 17 435). On average, 82.73% of known protein-coding genes were detected in ss&rd_RNA. In contrast, rd_RNA only covered 26.84% of genes, and tot_RNA covered 51.54% (Figure 1F). Particularly, ss&rd_RNA excelled in detecting low-abundance genes. Taking ss&rd_RNA_1 as an example, 1 305 genes were exclusively detected in this library but not in the tot_RNA or rd_RNA libraries, the median coverage of which was four (Figure 1G). Conclusively, consecutive size selection and rRNA depletion significantly improved bacterial RNA’s sequencing and mapping results.

### Nanopore DRS allowed the identification of intricate transcriptomic features

As Nanopore DRS eliminates the need to convert RNA to cDNA, it reduces biases introduced during library preparation. Moreover, it enables the detection of full-length RNA molecules, thereby providing a favorable alternative to studying the complex bacterial transcriptomes (41). Here, exemplary analyses were conducted on five DRS datasets generated from differentially processed *E. coli* RNA samples (Figure 1A). Without prior enrichment or treatment (8), both primary and processed transcripts were sequenced, thereby generating extensive transcript heterogeneity. For instance, in the ss&rd_RNA_1 sample, 11 types of transcripts were mapped to the *tff-rpsB-tsf* operon and its neighboring regions (Figure 2A–C). RNA boundaries were also easily obtained from the sequencing data, and multiple enriched transcript ends were observed (Figure 2A and B). It was seen that most reads covering the *tff-rpsB-tsf* operon started from the primary 5′ end and were terminated step-wise (Figure 2A–C). Consistent with a previous study (8), a few transcripts elongated their 3′ ends from the *tff-rpsB-tsf* operon to adjacent *pyrH* and *frr* genes (Figure 2C). Processed transcripts were also observed, as they lacked the 5′ fragment encoding the *tff* sRNA (Figure 2C). These findings potentially assist in updating gene annotations, mapping transcript boundaries, and identifying regulatory features.

Owing to the high coverage depth and long sequencing length (Figure 1B–F), ss&rd_RNA outperformed tot_RNA and rd_RNA in capturing high-resolution transcriptomic features. Across the whole *E. coli* transcriptome, the ss&rd_RNA_1 and ss&rd_RNA_2 datasets detected 3395 and 4317 transcript types, respectively. Each transcript type was supported by more than ten reads. By contrast, the numbers for rd_RNA and tot_RNA were 431 and 378 (Figure 2D). In support, more genes showed up in multiple transcriptional contexts in ss&rd_RNA than in tot_RNA and rd_RNA (Figure 2E). Compared with the complete list of transcriptional units deposited in the RegulonDB database (42), the ss&rd_RNA DRS reads increased the proportion of genes existing in at least two transcriptional context by >20% (Figure 2E). Based on the ss&rd_RNA_1 dataset, the boundaries of 225 operons recorded in RegulonDB (total = 2516) were extended (Supplementary Table S3), redefining the gene contents of these operons. A previous study applied PacBio long-read sequencing technology to decode the *E. coli* primary transcripts, termed SMRT-Cappable-seq (8). It identified 2369 and 2277 genes in individual samples. In the pooled dataset (*n* = 2665), about 45% of genes existed in at least two transcriptional contexts (Figure 2E). In comparison, the combined sequencing data of our two ss&rd_RNA samples revealed the transcriptional contexts of 2 987 genes, including the majority of genes detected by SMRT-Cappable-seq (2280 out of 2665) (Supplementary Figure S3). Approximately half of these genes were identified in more than one context (Figure 2E).

The same sequencing strategy, size selection combined with rRNA depletion and polyadenylation before DRS, was applied to the *S. aureus* total RNA (Table 1), and 2029 transcript types were identified. Transcripts aligned to the gene region encoding *rpsB* and *tsf* were exemplified in Supplementary Figure S4. Unlike the *tff-rpsB-tsf* operon transcripts in *E. coli*, none of the transcripts in *S. aureus* extended into the neighboring *pyrH* and *frr* genes. Compared with the experimentally and computationally determined operon structures in *S. aureus* strain MSSA476 (total = 1422) (43), the ss&rd_RNA DRS dataset in the present study stretched the boundaries of 89 operons (Supplementary Table S4). The in-house script used for operon and transcriptional context identification is deposited at https://github.com/JeremyQuo/ONT_DRS_bacteria_script.

In the ss&rd_RNA samples, many non-coding RNAs (ncRNAs) should have been lost due to the size selection step (Supplementary Figure S1), the mean size of which is 132 nt. Nevertheless, the ss&rd_RNA datasets detected more ncRNA reads than the tot_RNA and rd_RNA samples because of the increased total yield, covering more ncRNA genes (Supplementary Figure S5). Meanwhile, more mRNA molecules were sequenced in ss&rd_RNA than in tot_RNA and rd_RNA (Figure 1D), allowing more reliable quantification analysis of mRNA expression levels. Specifically, Spearman's rank correlation coefficient between the ss&rd_RNA replicates was 0.90, while the number calculated for the two rd_RNA samples was 0.73. The ss&rd_RNA datasets also correlated better with the NGS RNA-seq data than tot_RNA and rd_RNA (Figure 2F). In conclusion, DRS of ss&rd_RNA is a promising strategy for understanding complicated bacterial transcriptome architectures and reliably quantifying protein-coding gene expressions.

### Transcriptome-wide modification-free and m6A-modified IVT RNAs were generated as negative and positive controls

Multiple computational tools have been developed to detect RNA modifications based on Nanopore DRS. Some specifically detect m6A sites within the DRACH/RRACH motif and do not require a control sample (28,29). However, these approaches do not apply to non-eukaryotic epitranscriptomes (44) since the DRACH/RRACH motif is restricted to eukaryotic m6A modifications (45). Some other computational tools compare the differences between the sample of interest and its counterparts deprived of specific modifications to infer the modified sites (30–36), utilizing either current signal features or basecalling errors. Theoretically, these comparative methods can be applied to detect any modifications and decipher unknown epitranscriptomes.

In the present study, to enable the application of comparative computational tools, transcriptome-wide modification-free RNAs (IVT_neg) were synthesized via *in vitro* transcription for *E. coli and S. aureus* (Figure 3A). Meanwhile, a positive control sample containing exclusively m6A in the *E. coli* IVT transcriptome (IVT_pos) was prepared (Figure 3A). These IVT RNA samples were reported for the first time in the context of bacterial transcriptomes. Compared with the native ss&rd_RNA sequencing results, DRS of IVT_neg RNAs yielded fewer but adequate throughputs (Table 1 and Figure 3B). The two IVT_neg datasets covered most genes detected in the native samples (Figure 3C). On the other hand, sequencing of the *E. coli* IVT_pos sample generated far fewer reads and bases, and many of them cannot be aligned to the reference genome (Figure 3B). As a result, IVT_pos covered only 21.25% of genes sequenced in ss&rd_RNA_1 (Figure 3C). mRNA-mapping reads were specifically examined, with IVT_pos showing significantly lower amounts and shorter read lengths than other samples (Table 2). Regarding gene expression correlation, Spearman's coefficients calculated for IVT_neg and native samples were 0.81 and 0.80 in *E. coli* and *S. aureus*, respectively (Figure 3D). In comparison, the coefficient between IVT_pos and native RNAs was 0.70 (Figure 3D).

**Table 2.** Comparison of mRNA-mapping read features between native and *in vitro* transcribed (IVT) RNA libraries based on Dorado basecalling outputs

	*E. coli* ss&rd_RNA_1	*E. coli* ss&rd_RNA_2	*E. coli* IVT_neg	*E. coli* IVT_pos	*S. aureus* ss&rd_RNA	*S. aureus* IVT_neg
Read number	360 951	678 877	304 790	76 360	410 238	311 043
Base number	300 817 273	473 717 536	133 223 478	21 928 365	249 414 732	214 139 170
Average length	833.4	697.8	437.1	287.2	608	688.5
Median length	663	539	392	267	488	521
N50 length	1039	911	483	295	675	851
Median Q score	13.94	16.52	11.86	11.88	12.86	14.51
Covered gene number	3444	3651	2740	746	2092	2324

Read metrics of each dataset were further evaluated based on either Dorado or Guppy basecalling outputs. It is generally accepted that the presence of m6A can cause electric current variability, leading to the enrichment of basecalling errors in the modified reads compared with their unmodified equivalent (27). However, according to the Dorado basecalling results, the *E. coli* native sample, which contains m6A modifications at a level comparable to eukaryotes (15), displayed a higher read accuracy than its negative counterpart (Figure 3E). The IVT_neg samples demonstrated a more frequent occurrence of mismatches and indels (Figure 3E). Moreover, the quality scores of *E. coli* native reads were higher than those of IVT_neg reads (Figure 3E). In the case of *S. aureus*, the unmodified IVT RNAs also did not show any superiority across multiple read metrics compared with the native sample (Figure 3E). Instead, a different scenario emerged when Guppy basecalling results were used for assessment. Consistent with the common view, the *E. coli* and *S. aureus* IVT_neg samples showed higher read accuracies and quality scores than their native counterparts (Figure 3F). These findings highlighted the necessity of applying Guppy outputs for analyses when using error-based computational tools to infer modification sites. On the other hand, the possible reason for the Dorado results is that the new rna002_70bps_hac@v3 model improved the basecalling accuracy of modified nucleotides.

Additionally, regardless of using Dorado or Guppy for basecalling, the *E. coli* IVT_pos reads exhibited relatively lower or similar error rates compared with the negative control in terms of mismatches and indels (Figure 3E and F), which was inconsistent with the consensus (46,47). It is notable that most reads in the IVT_pos sample failed to align (Figure 3B) and thereby were excluded from the read accuracy analysis. This incomplete inclusion of sequencing outputs may partially explain the observed abnormalities. Meanwhile, the IVT_pos reads indeed showed poorer quality scores than the unmodified equivalents. These controversial results regarding IVT_pos read metrics underlined extra cautions that should be taken utilizing computational tools to identify modification sites.

### Comparative computational tools unveiled potential modification sites in the bacterial transcriptomes

**Figure 4.**
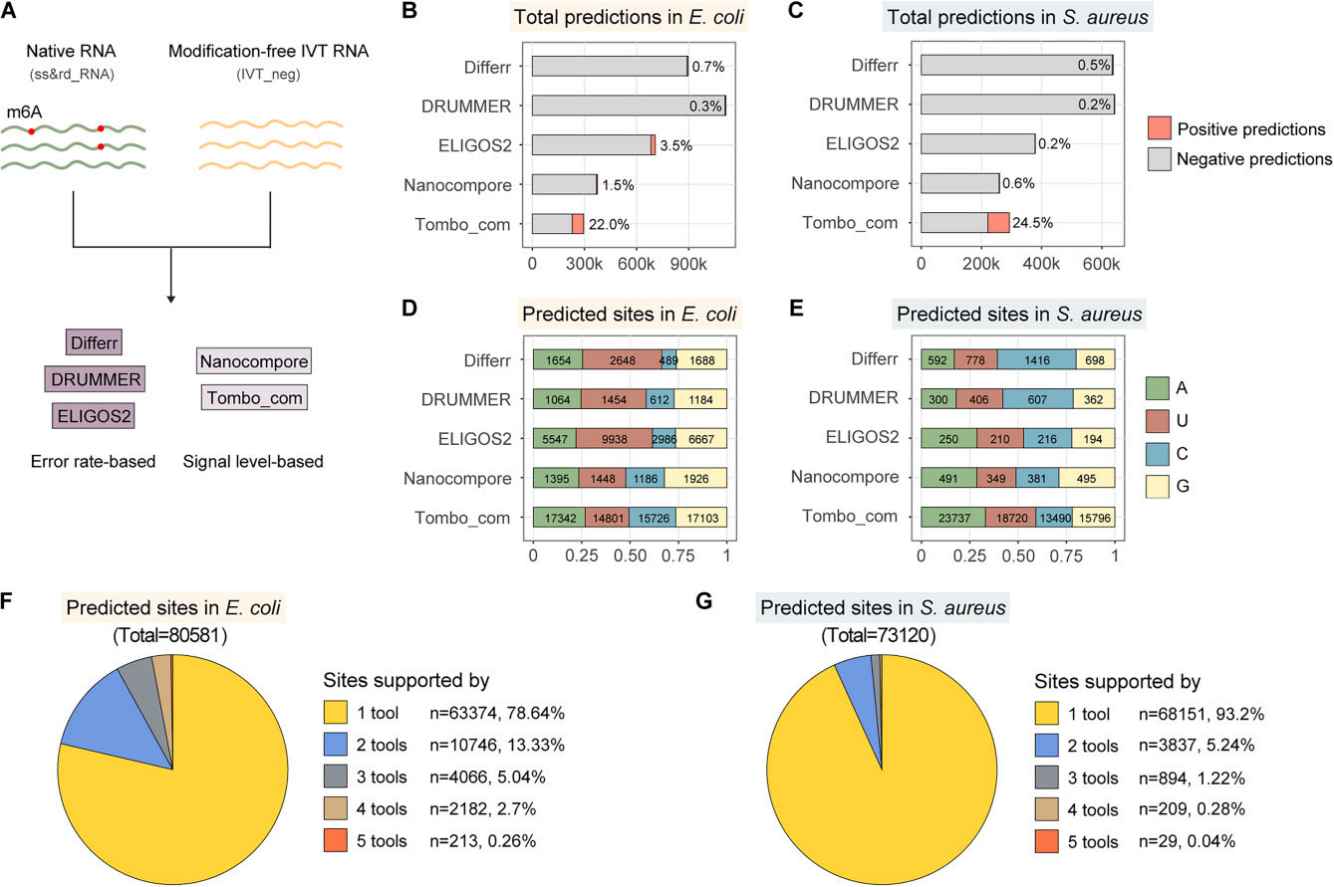
Bacterial epitranscriptome analysis using computational methods based on ONT DRS data. (**A**) Overview of the analysis workflow. ss&rd_RNA and IVT_neg samples were prepared and sequenced. The resulting DRS data were analyzed to identify modification sites based on either error rate-based (Differr, DRUMMER, and ELIGOS2) or signal level-based (Nanocompore and Tombo_com) methods. **(B**, **C)** Summary of prediction outputs of different computational tools in *E. coli* and *S. aureus*. The height of each bar indicates the predictable sites by individual methods. The number above each bar indicates the ratio of positive predictions against total predictions. **(D**, **E)** Contents of positive predictions by individual computational tools in *E. coli* and *S. aureus*. Numbers embedded in the bars indicate the amount of each type of modified nucleotide. **(F**, **G)** Percentage of modification sites identified by multiple tools in *E.coli* and *S. aureus*.

The Nanopore DRS data of native ss&rd_RNA and modification-free IVT_neg samples were analyzed using comparative computational tools to identify potential modification sites in the *E. coli* and *S. aureus* transcriptomes (Figure 4A). Error rate-based Differr, DRUMMER, ELIGOS2 and EpiNano_Error, as well as signal level-based Nanocompore, Tombo_com, and xPore, were applied. Since the error-based tools were developed based on the Guppy basecalling mistakes, Guppy outputs were employed for these methods. On the other hand, signal-based analyses were performed using the Dorado basecalling results, enabling a more precise assignment of raw current signals to individual nucleotides. Notably, EpiNano_Error did not generate any predictions, and xPore failed to output transcriptome-wide results due to an intrinsic issue (Supplementary Figure S6). Therefore, these two tools were excluded from downstream analyses.

In the *E. coli* ss&rd_RNA_1 and IVT_neg samples, 1 984 955 and 1 402 850 sites had a coverage depth greater than ten. In *S. aureus*, the numbers were 961 203 and 1 238 009 for native and unmodified RNAs, respectively. However, the predictable sites of each computational tool only accounted for a handful of these mapped sites, and error-based methods generally detected more sites than signal-based approaches (Figure 4B and C). Consistent with previous studies (44,48), respective computational methods differed dramatically in outputting modification sites (Figure 4B and C). Tombo_com generated the most abundant positive predictions, with 22% and 24.5% of sites reported as modified in *E. coli* and *S. aureus*, respectively. By contrast, only 0.2% and 0.3% of predictable sites were recognized as modified by DRUMMER.

The contents of predicted modification sites greatly varied between *E. coli* and *S. aureus* (Figure 4D and E). In *E. coli*, a considerable proportion (greater than 33.70%) of modifications were inferred by error-based methods to be present on uridines, while less than 14.19% were identified on modified cytidines. In contrast, with the same error-based approaches, decorated cytidines outnumbered uridines in *S. aureus*. Although these predictions might not reflect the actual modification profiles in *E. coli* and *S. aureus* since RNA modifications alter the current signals locally (34), they underscored the epitranscriptome differences between the two bacteria. It is further seen from the modification compositions that individual computational methods exhibited substantial variances in predicting modified nucleotides for the same bacterium (Figure 4D and E). Since integrating predictions from multiple tools could improve effectiveness (48), intersections between different prediction results were analyzed (Figure 4F, G and Supplementary Figure S7). Relatively considerable overlaps were observed in *E. coli*, with 21.36% of total predictions supported by at least two computational methods (Figure 4F). This ratio decreased to 6.80% in the case of *S. aureus* (Figure 4G), suggesting that more modification sites could be confidently identified in *E. coli* than in *S. aureus*.

### High-confidence m6A sites were identified with the assistance of MeRIP-Seq

**Figure 5.**
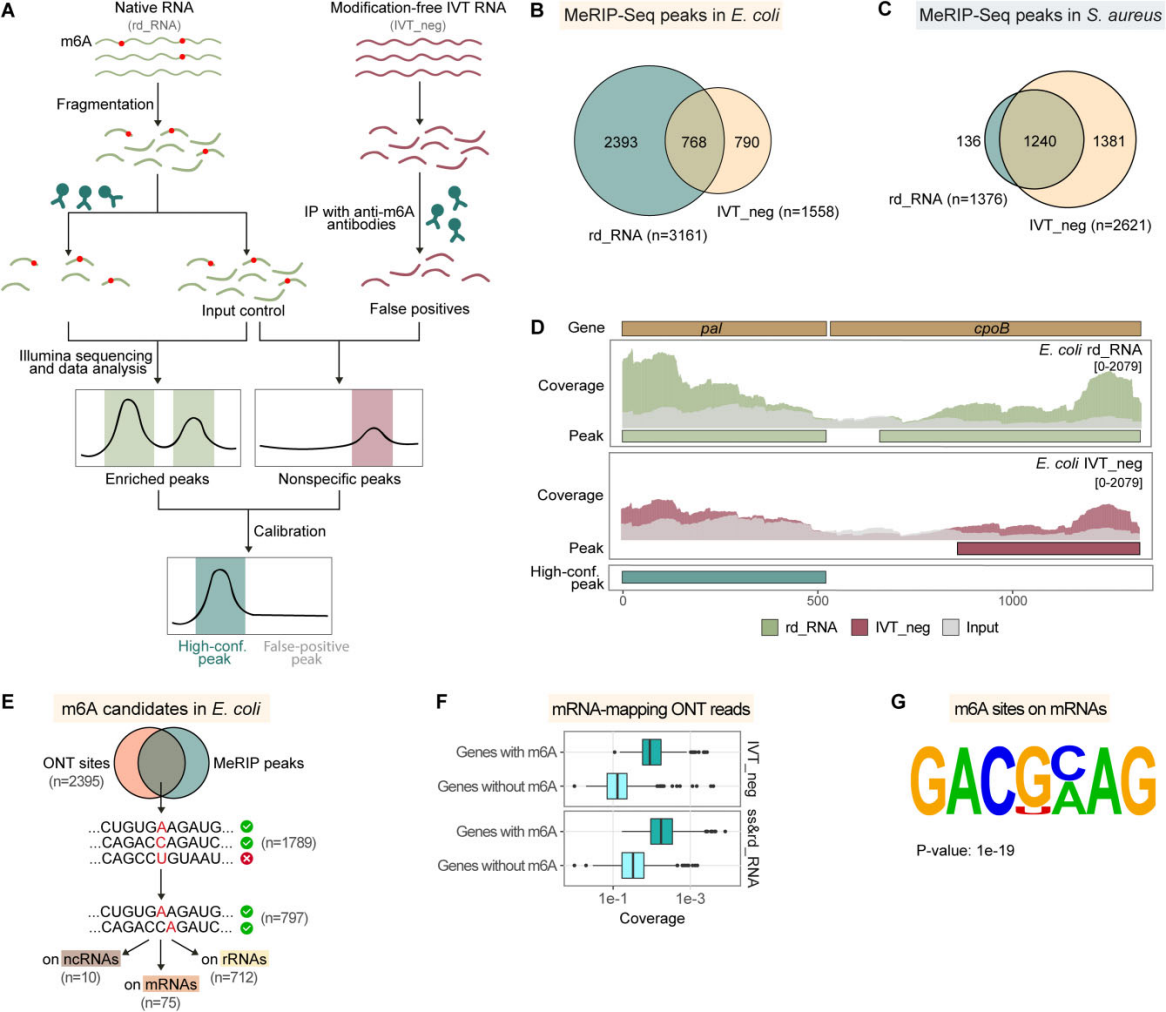
Profiling of m6A sites in bacterial transcriptomes with the assistance of Methylated RNA Immunoprecipitation Sequencing (MeRIP-Seq). (**A**) MeRIP-Seq workflow utilizing modification-free RNA libraries for calibration. Unmodified IVT_neg samples were processed in parallel with native rd_RNA samples. The putative m6A peaks in the IVT sample were used to calibrate the m6A peak in the native sample to generate high-confidence results. (**B**) Venn diagram showing the peaks in native and modification-free RNA libraries in *E. coli*. The MeRIP sequencing data was processed using exomePeak2. (**C**) Venn diagram showing the peaks in native and modification-free RNA libraries in *S. aureus*. (**D**) Representative gene loci exemplifying the calibration principle. (**E**) Schematic of m6A profiling process combining ONT and MeRIP results in *E. coli*. First, ONT sites located within MeRIP peaks were extracted from total predictions. Amongst these sites, adenosines, either reported as modified themselves or within two-base distances of predicted uridines/cytosines/guanines, were redeemed as m6A candidates. (**F**) Sequencing depth of mRNA-mapping ONT reads in the IVT_neg and ss&rd_RNA datasets. Genes with m6A indicate genes containing high-confidence m6A sites identified in (E). (**G**) Leading motif at putative m6A positions on *E. coli* mRNAs. The motif was identified using Homer.

It is feasible to infer the presence of eukaryotic m6A modifications by comparing native and unmodified IVT RNAs using Nanopore computational tools since m6A modifications in eukaryotic mRNAs are largely restrained to the DRACH/RRACH motifs (45). The error rate or signal changes within these motifs are most likely caused by modified adenosines. However, the motif preference of m6A modifications in prokaryotic cells remains unclear, making it challenging to assign m6A sites solely by comparing native and unmodified IVT RNAs. Therefore, MeRIP-Seq, the most common method for m6A mapping (49), was performed to aid computational approaches to identify m6A modifications specifically. To improve reliability, modification-free RNA libraries were processed in parallel with the native RNA samples (21). The putative m6A peaks in the native transcriptomes were calibrated using IVT peaks to generate high-confidence results (Figure 5A). This way, 2 393 m6A peaks were identified in *E. coli* (Figure 5B), covering 14% of the total gene length, with a median length of 150 nt and an average length of 250 nt. In contrast, only 136 peaks were maintained in *S. aureus* after calibration (Figure 5C), in consistency with a previous study reporting a significantly lower m6A/A ratio in *S. aureus* compared with *E. coli* (15). The majority of the detected *E. coli* m6A peaks were located on the mRNA molecules (Supplementary Figure S8), as exemplified in Figure 5D.

In *E. coli*, 51.76% of modification sites detected by at least one Nanopore computational tool were located within the MeRIP peaks (Supplementary Figure S9). In comparison, the ratio of Nanopore-detected sites within the MeRIP peaks was 1.24% in *S. aureus* (Supplementary Figure S9), partially due to a limited number of peaks called in the *S. aureus* transcriptome (Figure 5C). Two nucleotides in the *E. coli* 23S rRNA, A1618 and A2030, have been verified to be modified with m6A (50,51). Here, the calibrated MeRIP peaks covered these regions (Supplementary Figure S10). Meanwhile, A1616, neighboring A1618, and A2030 were repeatedly detected by four Nanopore-based approaches (Supplementary Figure S10). Therefore, sites within MeRIP peaks and supported by at least four tools were considered high-confidence. A total of 1789 such positions were identified in the *E. coli* transcriptome accordingly, whereas none were detected in *S. aureus*. Adenosines were specifically extracted from the prediction result and defined as putative m6A sites (Figure 5E). Considering that m6A modifications can alter the current signals of neighboring nucleotides (34), adenosines within two-base distances of predicted uridine/cytosine/guanine sites were also regarded as m6A candidates (Figure 5E). This way, 797 sites were obtained, including 712 on rRNAs, 75 on mRNAs and 10 on ncRNAs (Supplementary Tables S5-S7).

The detected mRNA m6A modifications were located on 21 genes (Table 3 and Supplementary Table S3). Via GO analysis (Supplementary Tables S8-S10), these m6A-modified genes were primarily of RNA or protein binding functions and associated with protein unfolding, translational elongation, transcription antitermination, ribosomal small subunit assembly, etc. Notably, the number of final mRNA m6A predictions was minimal compared to the 2267 MeRIP peaks on mRNAs (Supplementary Figure S8). The reason can be that in the context of Nanopore DRS detections, only the regions covered by adequate reads are predictable. On the other hand, MeRIP-Seq benefits from a high sensitivity due to the application of NGS RNA-seq. In corroboration with this hypothesis, Nanopore DRS reads covering high-confidence m6A sites were shown to accommodate significantly higher coverage depth than those null of such sites (Figure 5F). The identified mRNA m6As were subsequently subjected to Homer motif analysis, and three motifs were significantly enriched (Supplementary Figure S11). The leading consensus sequence is GACGCMAG (M = C/A, *P*-value = 1e-19) (Figure 5G), found at 16.00% of putative m6A positions on mRNAs. The 10 potential ncRNA m6A sites were found on two tRNAs (*lysY* and *valT*) and one small RNA (*raiZ*) (Supplementary Table S4).

**Table 3.** High-confidence m6A sites on the *E. coli* mRNA molecules

Gene name	Gene length	m6A position
aspA	1437	425
cspA	213	27, 30, 82, 83, 109, 113, 119, 137, 140, 141, 146, 180, 205, 212
cspE	210	7
dnaK	1917	844
gatY	855	734
glpD	1506	962, 1340
groL	1647	331, 673, 1433, 1583, 1596, 1639
hflX	1281	148
ompC	1104	167
pal	522	425
proX	993	835, 866
raiA	342	6, 138, 200, 206, 212, 214, 223
rpmF	174	9
rpoA	990	250, 871
rpsA	1674	1088, 1268, 1272, 1324, 1363, 1457, 1516, 1571, 1613, 1616
rpsG	540	534
rpsU	216	1
secY	1332	23
tig	1299	1289
tufA	1185	263, 545, 758, 800
tufB	1185	181, 268, 479, 545, 676, 709, 732, 733, 751, 755, 803, 924, 940, 941, 942, 947

In addition, numerous m6A candidates were assigned to fourteen 16S and 23S rRNA paralogs. Unlike mRNA molecules, *E. coli* rRNAs are extensively decorated for full functionality. Thirty-six modified nucleotides have been confirmed, including twenty-three methylated, ten pseudouridines, one methylated pseudouridine, one dihydrouridine, and one hydroxycytidine (52). Given this high modification density and diversity, combined with the wide MeRIP peak ranges on rRNAs, the m6A profiling procedure (Figure 5E) inevitably mistreated many positions as m6A modifications. To depict a complete picture of rRNA modifications in *E. coli*, all rRNA sites supported by at least four computational tools (n = 1 737) were retained and placed in corresponding gene positions (Supplementary Figure S12). Many known modified nucleotides were covered or surrounded by Nanopore predictions (Supplementary Figure S12), indicating the reliability of applying computational tools to identify RNA modifications. More specifically, 18 out of 25 modification sites on 23S rRNA and 6 out of 11 modification sites on 16S rRNA were detected by Nanopore methods. On the other hand, a considerable number of predicted sites clustered in regions null of any verified modifications (Supplementary Figure S12), suggesting that the abundance of modifications on rRNAs may be higher than expected. Moreover, the modification patterns slightly varied across different copies of 16S or 23S rRNA molecules (Supplementary Figure S12), which warrants further investigation.

### Nanopore DRS of m6A-modified IVT RNA corroborated the feasibility of computational analysis of m6A modifications

**Figure 6.**
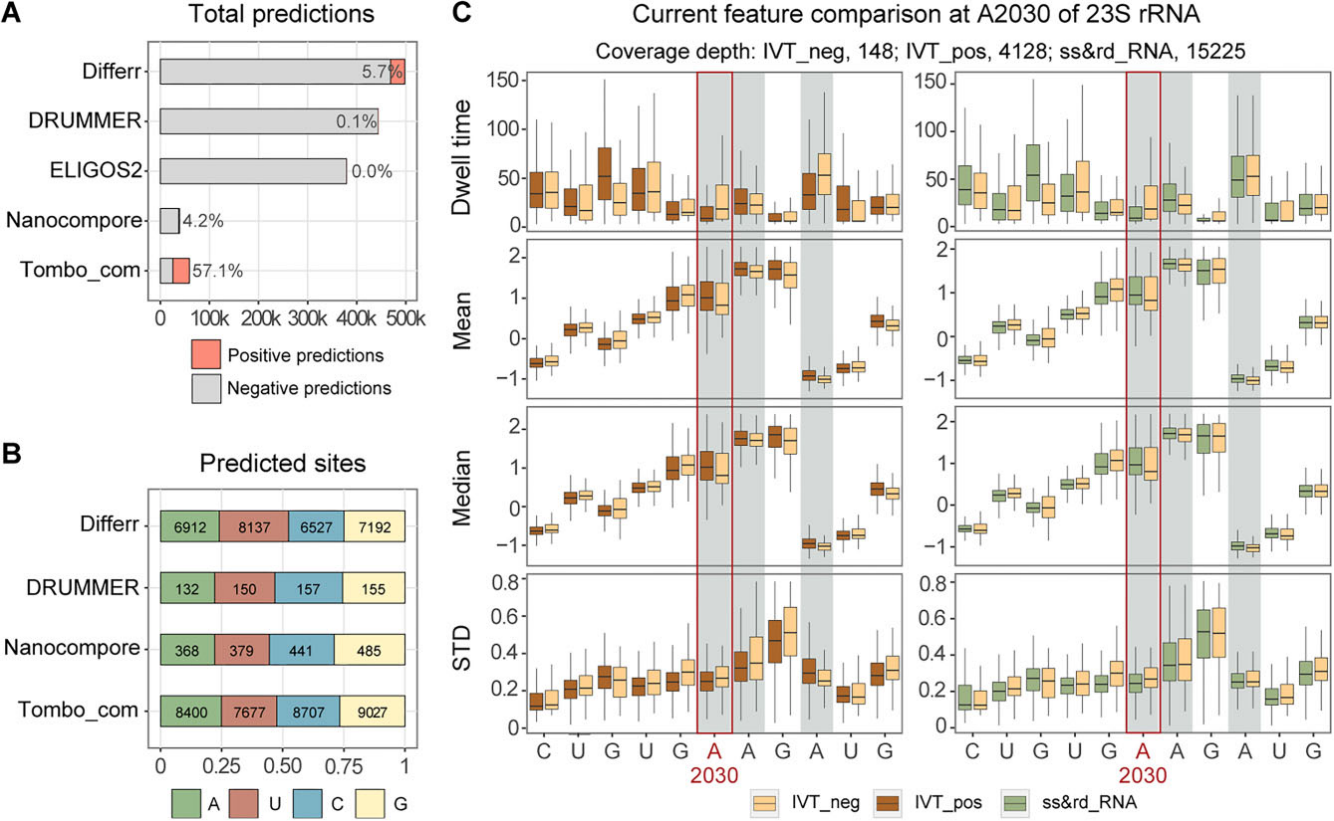
Analysis of m6A-modified transcriptome-wide IVT RNA library. (**A**) Summary of prediction outputs of different computational tools. (**B**) Contents of positive predictions by individual computational tools. (**C**) Current features of different samples at A2030 and its neighboring region of *E. coli* 23S rRNA. Dwell time, mean intensity, median intensity, and standard deviation (STD) were depicted for modification-free IVT_neg, m6A-modified IVT_pos, and native ss&rd_RNA at each position. nanoCEM was used for plotting.

An m6A-modified IVT RNA sample (IVT_pos), where adenosines were replaced entirely with m6A, was prepared as a positive control for mapping m6A in native RNAs. Sequencing of this sample yielded very few reads and bases (Figure 3B). A limited proportion of bases (36.68%) were aligned to the *E. coli* genome (Figure 3B), and only 176 832 positions were covered by at least ten reads. As a result, a handful of sites were predictable by individual computational tools (Figure 6A). Amongst the tested positions, the positive prediction proportions for Differr, DRUMMER, ELIGOS2 and Nanocompore ranged from 0.0–5.7%. Since m6A accounted for approximately a quarter of the total bases, these values were considered extremely low, suggesting limited recall of these tools, which might result from low coverage depth and incomplete read alignment. The predicted modification sites were also found not to be restricted to adenosines. Instead, they were almost evenly attributed to four types of bases (Figure 6B). The primary reason is that a modification can alter the current signals locally, and intensity shifts at modified positions sometimes spread to adjacent k-mers (34,53). Therefore, all bases should be inspected regardless of the detected modification types.

Due to the limited sequencing throughput and low-quality alignment, very few positions in the IVT_pos sample were communally detected by different computational methods (Supplementary Figure S13). Moreover, the IVT_pos reads did not show higher basecalling error rates than their unmodified and native equivalents (Figure 3E and F), negatively affecting the application of error-based methods. As a result, the prediction outputs from individual computational methods showed a small proportion of intersections (Supplementary Figure S14). Nevertheless, the IVT_pos sample could serve as a reference for direct current signal comparisons. For instance, the current features of the 23S rRNA A2030 and its adjacent region were depicted for IVT_pos, IVT_neg, and native RNAs (Figure 6C). Consistent with the known m6A modification at A2030 in the native rRNA, the native and IVT_pos signals were indistinguishable at this position and the adjacent 2028U and 2029G. They resembled each other across multiple features, including dwell time, mean intensity, median intensity, and standard deviation (STD). However, at A2031 and A2033, IVT_pos exhibited nonnegligible differences from the native RNA signals, complying with the fact that these sites were decorated with m6A in IVT_pos while unmodified in the native RNA.

## Discussion

It has come to an awareness that bacterial gene expression is a complex process involving extensive regulatory mechanisms. Much relevant knowledge is acquired from the model organism *E. coli* (54) and several bacterial pathogens such as *Listeria monocytogenes* (55). The growing interest in human microbiota and environmental microbial communities has highlighted the importance of various previously understudied bacterial species. Approaches that can rapidly and comprehensively analyze the RNA biology of a bacterium of interest are needed. In this regard, Nanopore DRS provides a promising platform for the simultaneous identification of intricate transcriptomic features and various RNA modifications.

To exert the full potential of Nanopore DRS in characterizing bacterial transcriptomes and epitranscriptomes, an RNA preprocessing procedure was introduced in the present study for improved sequencing and mapping quality. Gram-negative *E. coli* and gram-positive *S. aureus* RNAs were used for illustration. Briefly, total RNAs isolated from bacterial cells were subjected to size selection to remove highly structured small-sized RNA molecules, followed by rRNA depletion and polyadenylation (Figure 1A). This way, the total sequencing throughput of the processed bacterial RNA samples (ss&rd_RNA) increased to approximately twice that of the total RNA samples (tot_RNA) and 19 times that of solely rRNA-depleted samples (rd_RNA) (Figure 1B). Moreover, the ss&rd_RNA reads were of significantly longer length and higher quality (Figure 1C), which could be attributed to two reasons. First, ncRNA reads, depleted in the ss&rd_RNA samples, had the lowest read qualities and shortest read lengths than mRNA and rRNA reads (Supplementary Figure S15). Second, unmapped reads accounted for a large portion of the tot_RNA and rd_RNA datasets and showed inferior Q scores and read lengths compared with mapped reads (Supplementary Figure S16). The broad existence of low-quality reads in the tot_RNA and rd_RNA samples potentially interferes with the sequencing procedure of Nanopore DRS, resulting in poorer overall sequencing quality. The improvements in sequencing and mapping quality consequently facilitated the high-resolution characterization of bacterial transcriptome architectures, as exemplified by the *E. coli* ss&rd_RNA samples (Figures 1D–F and 2A–E). For example, the ss&rd_RNA_1 dataset covered 3444 of 4288 known protein-coding genes and identified 3395 types of primary and processed transcripts. Nearly half of the *E. coli* genes were detected in at least two transcript types, consolidating the complexity of bacterial transcriptomes. The ss&rd_RNA data can also be used for quantitative analysis, as it showed a reasonable correlation with the NGS RNA-seq data regarding protein-coding gene expression levels (Figure 2F). In additional, despite the removal of small-sized RNAs (Supplementary Figure S1), the ss&rd_RNA datasets contained more ncRNA reads than the tot_RNA and rd_RNA samples and showed higher coverages (Supplementary Figure S5).

In addition to transcriptome analysis, Nanopore DRS data allows the detection of RNA modifications existing on various RNA molecules. The general understanding of bacterial RNA modifications is primarily based on investigations on rRNAs and tRNAs (52,56). Only a few studies globally mapped RNA modifications on bacterial mRNAs (15); however, no single-nucleotide resolution has been realized. The present research prepared and sequenced two transcriptome-wide modification-free IVT RNA libraries, which served as negative controls (Figure 3). They and their native equivalents were analyzed by seven Nanopore-based comparative computational tools to identify potential modification sites (Figure 4). This way, all kinds of modified nucleotides were recognized but failed to define a specific chemical modification. Therefore, two m6A-detecting NGS-based methods, including MeRIP-Seq and m6A-REF-seq, were performed to filter m6A candidates from the total computational outputs, finally generating single-nucleotide resolution m6A predictions (Figure 5). Notably, although MeRIP is a well-established and the most widely-used method for global detection of m6A, there are concerns about its limited reproducibility (49) and false-positive peak calling (21). In the present study, an IVT_neg sample was processed in parallel to calibrate the MeRIP result, displaying a considerable number of false-positive peaks, with the majority located in the mRNA regions (Supplementary Figure S17). Additionally, the performance of different peak calling tools varied greatly (57). The MeRIP data in the present study was processed using two popular pipelines, Macs2 and exomePeak2. It was found that changes to Macs2 parameter settings dramatically altered peak calling results (Supplementary Figure S18). Generally, Macs2 yielded fewer but longer peaks than exomePeak2 (Supplementary Figure S18). In all cases, only the exomePeak2 pipeline showed significantly more m6A peaks in the native RNAs than in the IVT counterparts (Supplementary Figure S18). Considering that the exomePeak2 result also complied with the known fact that the *E. coli* transcriptome contains more m6A modifications than the *S. aureus* transcriptome (15), exomePeak2 was applied for formal peak calling analysis. In addition to MeRIP-Seq, m6A-REF-seq was conducted to identify m6A at the ACA motif (Supplementary Figure S19) (14). However, out of 28 921 ACA motifs in the *E. coli* transcriptome, only two positions were potentially modified with m6A after calibration using the unmodified IVT RNA dataset. Therefore, it is highlighted again that m6A modifications in *E. coli* significantly differed from those in eukaryotes. An alternative method termed GLORI uses glyoxal and nitrite-mediated deamination of unmethylated adenosines to quantify m6A, which conceptually resembles bisulfite-sequencing-based quantification of DNA 5-methylcytosine (58). This method is promising to be unbiased and convenient for the absolute quantification of the m6A methylome.

The modification sites predicted by different computational methods varied (Figure 4B-E). Those supported by at least four methods were considered high-confidence since two known m6A sites on the *E. coli* 23S rRNA were repeatedly detected by four tools. A total of 2395 and 238 positions met this standard in the *E. coli* and *S. aureus* transcriptomes, respectively (Figure 4F and G). Amongst these sites, 797 putatively modified adenosines were located within the MeRIP peaks in *E. coli*, 75 of which were on mRNAs covering 21 genes. In contrast to the small number of 75 mRNA m6A sites, MeRIP-Seq detected 2267 m6A peaks on mRNAs (Supplementary Figure S8), and it has been reported that the m6A/adenosine ratio is around 0.28% on *E. coli* mRNAs (15). Therefore, the m6A-modified positions were underestimated in the present study. The primary reason is that a stringent filter was imposed to obtain high-confidence sites from the total computational predictions. Only those detected by at least four tools were used for downstream analysis. However, the five computational methods applied different preset parameters to process the datasets. The number of measured sites greatly differed from 293 512 (for Tombo_com) to 1 109 819 (for DRUMMER) in *E. coli* (Supplementary Figure S13). A small subset of these sites, 320 974 in number, was assessed by at least four tools. In other words, many positions were ignored in this study, leading to a limited m6A identification on mRNAs. Coverage depth is another factor potentially affecting the prediction results. It not only determines whether a given position is included in the analysis but also impacts detection sensitivity. This hypothesis was consolidated by the finding that the final high-confidence m6A candidates were in the high-coverage regions (Figure 5F). Besides, stoichiometric estimation was disabled in the present study since intersections of different computational tool predictions were adopted while most tools cannot estimate the m6A/A ratio at modification sites.

Further Homer motif analysis indicated that the leading consensus sequence of *E. coli* mRNA m6A modifications is GACGCMAG (M = C/A, P-value = 1e-19) (Figure 5G), which was different from the former reported UGCCAG (15). The previous study applied photo-crosslinking-assisted MeRIP-seq to the rRNA-depleted RNAs of overnight cultured *E. coli*, identifying 265 m6A peaks with lengths around 23 nt (15). Among the numerous mRNA MeRIP peaks obtained in our study (*n* = 2267), only 25 overlapped with previously reported peaks. New ncRNA MeRIP peaks (*n* = 15) were also identified (Supplementary Table S11), showing no overlapping with the former study. Moreover, none of the high-confidence Nanopore-predicted m6A candidates fell into these reference regions. There are several explanations. First, the *E. coli* cells were harvested at the log phase (OD600 0.4–0.6) in the present study, while the previous research collected overnight cultured cells (15). It has been narrated that exponential-phase samples systematically differ from stationary-phase samples (59). Since RNA modifications are generally dynamically regulated (60), the bacterial m6A profiles may significantly change at different growth stages. The differed gene expressions may also impact the MeRIP-seq detection results. Second, the two studies used different tools for peak calling. exomePeak2 and Macs were applied, respectively. Even with the same dataset, the two tools outputted immensely varied results (Supplementary Figure S18). Therefore, the intrinsic biases of peak calling tools also contribute to the inconsistent m6A peaks in the two studies. At last, MeRIP-seq is a method with low reproducibility. In eukaryotic samples, m6A peak overlap in mRNAs varies from ∼30 to 60% between studies (49). This ratio can be lower for prokaryotic RNAs due to less valid sample preparation and data processing procedures.

In addition to 85 putative m6A candidates, numerous other modification sites (*n* = 1598) were supported by at least four computational tools in the *E. coli* RNA population, excluding rRNAs (Figures 4F and 5E). A study demonstrated the existence of multiple RNA modifications on the *E. coli* mRNA molecules via Liquid Chromatography with tandem mass spectrometry, such as 2′-O-methylation, 5-methylcytosine, pseudouridine, inosine, etc (16). The Nanopore-identified sites can be decorated with these modifications. However, the exact position and corresponding chemical alternation cannot be determined without further supporting information. One solution is to prepare a positive control sample containing a specific modification. By comparing the current signal features at designated positions, the native RNA locations showing the same signal patterns as the positive control equivalents can be identified as equipped with identical modifications. In this study, m6A-modified IVT RNAs (IVT_pos) were synthesized and sequenced (Figure 3A). Indeed, at 23S rRNA A2030 and adjacent positions, in particular U2028 and G2029, the native and IVT_pos samples generated indistinguishable values regarding dwell time, mean intensity, median intensity, and STD of current signals (Figure 6C). On the other hand, the IVT_pos sample exhibited extremely poor sequencing and mapping quality (Figure 3B and C). Of the 94.50 Mb sequencing data, only 36.68% were aligned to the *E. coli* genome in the case of Dorado basecalling. Furthermore, despite a decreased read quality, the basecalled IVT_pos reads showed a higher mapping accuracy than native and IVT_neg reads (Figure 3E and F). This abnormality, in combination with the inferior data quality, hindered further application of the IVT_pos dataset in the present study.

Collectively, this study demonstrated the potential of applying Nanopore DRS to quickly and systematically characterize bacterial transcriptomes and epitranscriptomes. The size selection step exerted before rRNA depletion and polyadenylation dramatically increased sequencing and mapping qualities, significantly facilitating downstream analyses. Intricate bacterial transcriptome signatures can be easily obtained with a high resolution. By comparing with a modification-free sample, putative modification sites were predicted by computational tools. MeRIP-Seq further assisted in identifying high-confidence m6A positions at a single-nucleotide resolution. These works are transferrable to other bacterial species, especially those understudied, which can advance the knowledge of complex bacterial gene expression regulatory networks. Nevertheless, a few limitations remain in the present study. In particular, different Nanopore-based modification-calling computational tools showed limited consistency with each other, raising concerns about their reliability. Moreover, the proposed size selection step removed small-sized RNA molecules, which is detrimental to the quantitative study of small regulatory RNAs. The new Nanopore DRS kit (SQK-RNA004) is being released and promises higher accuracy and efficiency. Its improvement may help address these problems and attract more interest in utilizing Nanopore DRS to investigate bacterial transcriptomes and epitranscriptomes conveniently.

## Supplementary Material

gkae601_Supplemental_Files

## Data Availability

All sequencing data generated in this article is deposited in the NCBI database with the link https://www.ncbi.nlm.nih.gov/bioproject/PRJNA1052951/. All analysis pipelines and custom scripts used for this manuscript are available at https://github.com/JeremyQuo/ONT_DRS_bacteria_script and https://zenodo.org/doi/10.5281/zenodo.11664595.
