# Supplementary material for: Analysis of bacterial transcriptome and epitranscriptome using nanopore direct RNA sequencing
Source: Nucleic Acids Res. 2024 Jul 16;52(15):8746–62. doi: 10.1093/nar/gkae601 (PMC11347139; doi:10.1093/nar/gkae601)
Supplement: gkae601_Supplemental_Files [file gkae601_supplemental_files.zip › supplementary_materials.pdf]

Table S1. Raw read characteristics and mapping statistics based on Guppy basecalling outputs.

	E. coli [1] tot_RNA	*E. coli* rd_RNA_1	*E. coli* rd_RNA_2	*E. coli* ss&rd_RNA_1	*E. coli* neg_RNA	*E. coli* pos_RNA	*S. aureus* [2] ss&rd_RNA	*S. aureus* neg_RNA
Read number	650,632	145,949	138,570	1,967,477	1,875,902	342,889	1,850,033	906,980
Base number	605,230,297	53,494,356	51,794,097	1,454,620,370	591,319,758	262,785,859	915,653,810	483,123,034
Average length	930	366.5	373.8	738	315	766	495	532
Median length	841	255	331	636	284	186	408	469
N50 length	1,375	489	447	913	356	6,052	597	606
Median Q score	6.96	10.47	9.75	8.47	9.28	8.81	9.15	10.99
Ratio of mapped reads (%)	51.13	67.97	88.04	81.95	78.29	49.91	76.94	89.91
Ratio of mapped bases (%)	67.87	51.88	85.93	82.80	74.56	13.00	82.50	89.89
Ratio of reads mapped to mRNA (%)	2.61	22.03	10.95	19.18	14.91	22.35	18.22	32.40
Ratio of bases mapped to mRNA (%)	2.54	14.98	9.32	18.05	15.34	5.46	17.87	34.82

[1] *E. coli*, *Escherichia coli*.

[2] *S. aureus*, *Staphylococcus aureus*.

Table S2. Comparison of mRNA-mapping read features between native and in vitro transcribed (IVT) RNA libraries based on Guppy basecalling outputs.

	E. coli ss&rd_RNA_1	*E. coli* neg_RNA	*E. coli* pos_RNA	*S. aureus* ss&rd_RNA	*S. aureus* neg_RNA
Read number	377,387	279,791	76,654	337,166	293,898
Base number	262,558,977	90,708,451	14,348,108	168,627,336	168,223,440
Average length	826	428	276	621	700
Median length	659	385	261	516	532
N50 length	1,035	479	299	722	879
Median Q score	8.76	9.53	11.03	9.90	10.31
Covered gene number	3472	2737	761	2092	2324

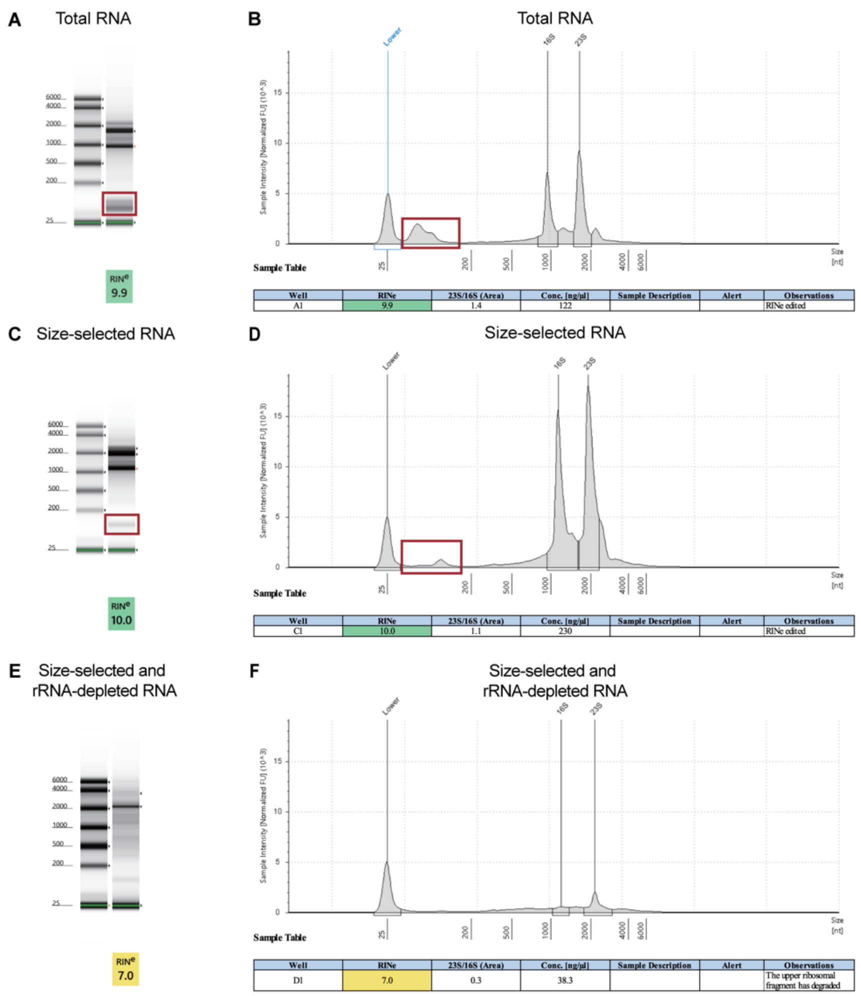

Figure S1. Changes in RNA size distribution during the ss&rd_RNA_2 sample pre-processing procedure. The 4200 TapeStation System was applied to analyze RNA size distribution. (A) and (B) Electropherogram and peak intensity plot of total RNA isolated from the *E. coli* K-12 cells. (C) and (D) Electropherogram and peak intensity plot of size-selected RNA. Size selection was performed using the SPRIselect Beads at a bead-to-sample volume ratio of 0.8. (E) and (F) Electropherogram and peak intensity plot of size-selected and rRNA-depleted RNA. rRNA depletion was performed using the RiboMinus™ Transcriptome Isolation Kit, bacteria.

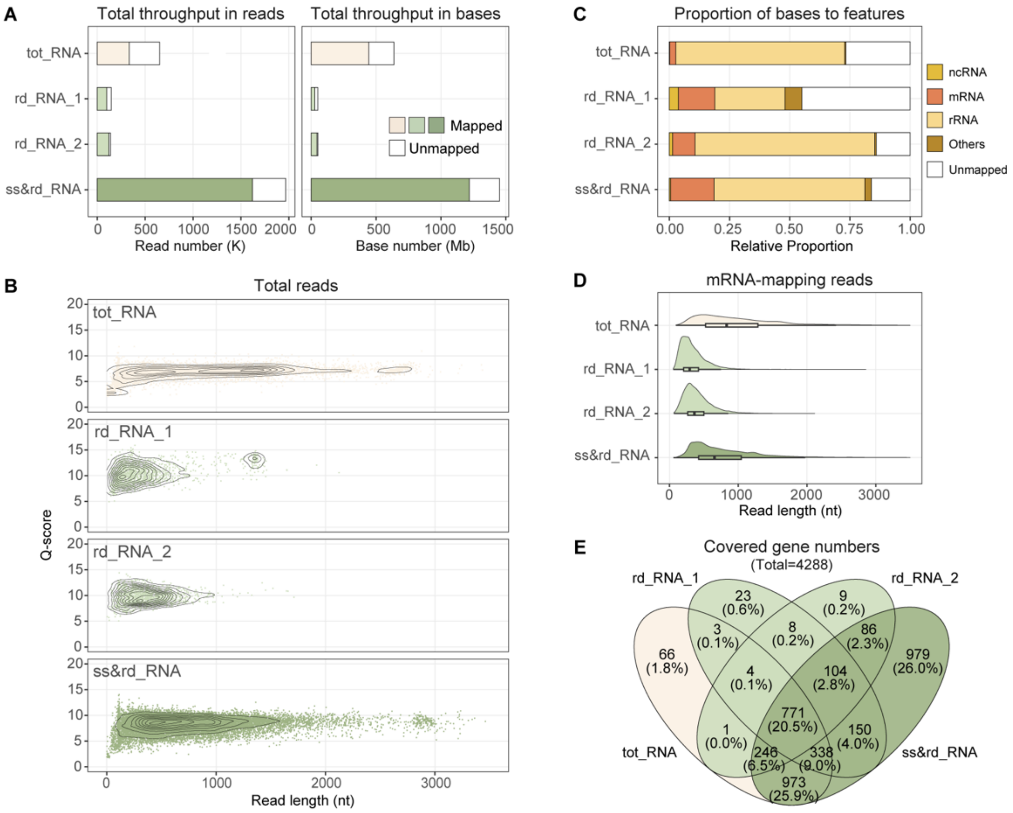

Figure S2. Raw read characteristics and analysis of mapped reads based on Guppy basecalling results. *E. coli* strain K12 total RNAs were isolated from cells grown to a log phase and subjected to Oxford Nanopore Technologies (ONT) Direct RNA Sequencing (DRS) following different processing procedures. (A) Total sequencing throughput. (B) Relationship between read length and read quality of raw reads. (C) Proportion of bases to annotation features. (D) Read length of mRNA-mapping reads. (E) Veen diagram showing the intersections of covered genes by individual datasets.

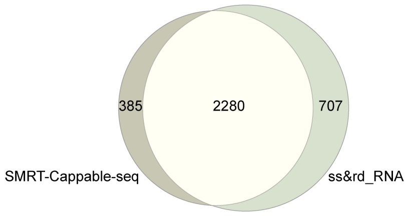

Figure S3. The intersection of genes identified by SMRT-Cappable-seq and ss&rd_RNA. The SMRT-Cappable-seq pooled dataset is referenced from (8). ss&rd_RNA_1 and ss&rd_RNA_2 sequencing results are pooled together for the analysis, and each gene is supported by at least ten reads.

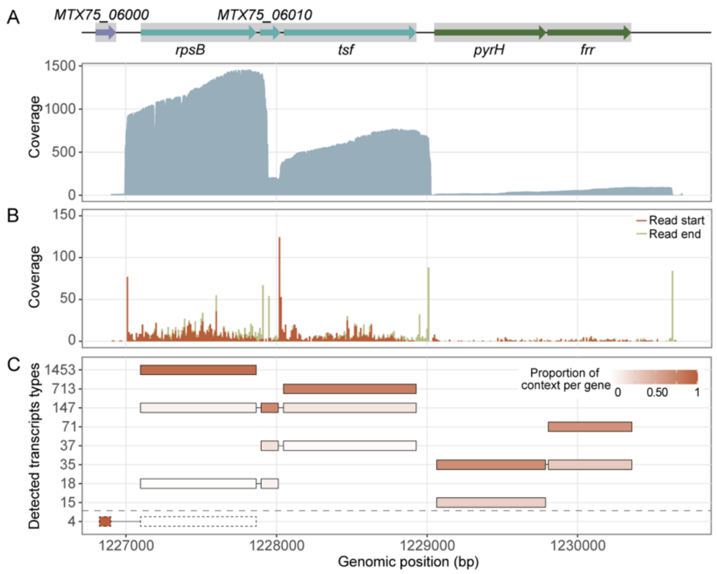

Figure S4. *S. aureus* transcriptomic features identified by ONT DRS. (A) Coverage plot of reads aligned to the gene region encoding *rpsB* and *tsf* in the ss&rd_RNA dataset. (B) Visualization of read boundaries. Bin size = 10. (C) Different types of transcripts identified in this region.

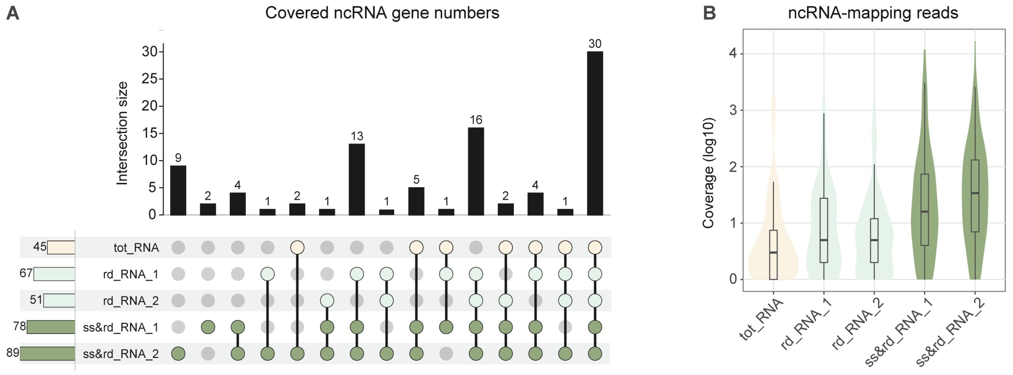

Figure S5. Analysis of ONT DRS ncRNA-mapping reads in the *E. coli* datasets. This analysis is based on the Dorado basecalling results. All the ncRNAs encoded in the *E. coli* genome (tRNA excluded) were included in the present analysis (n=98). (A) UpSet plot showing the intersections of covered ncRNA genes by individual datasets. (B) Violin plot showing the coverage distribution of ncRNA reads in each dataset.

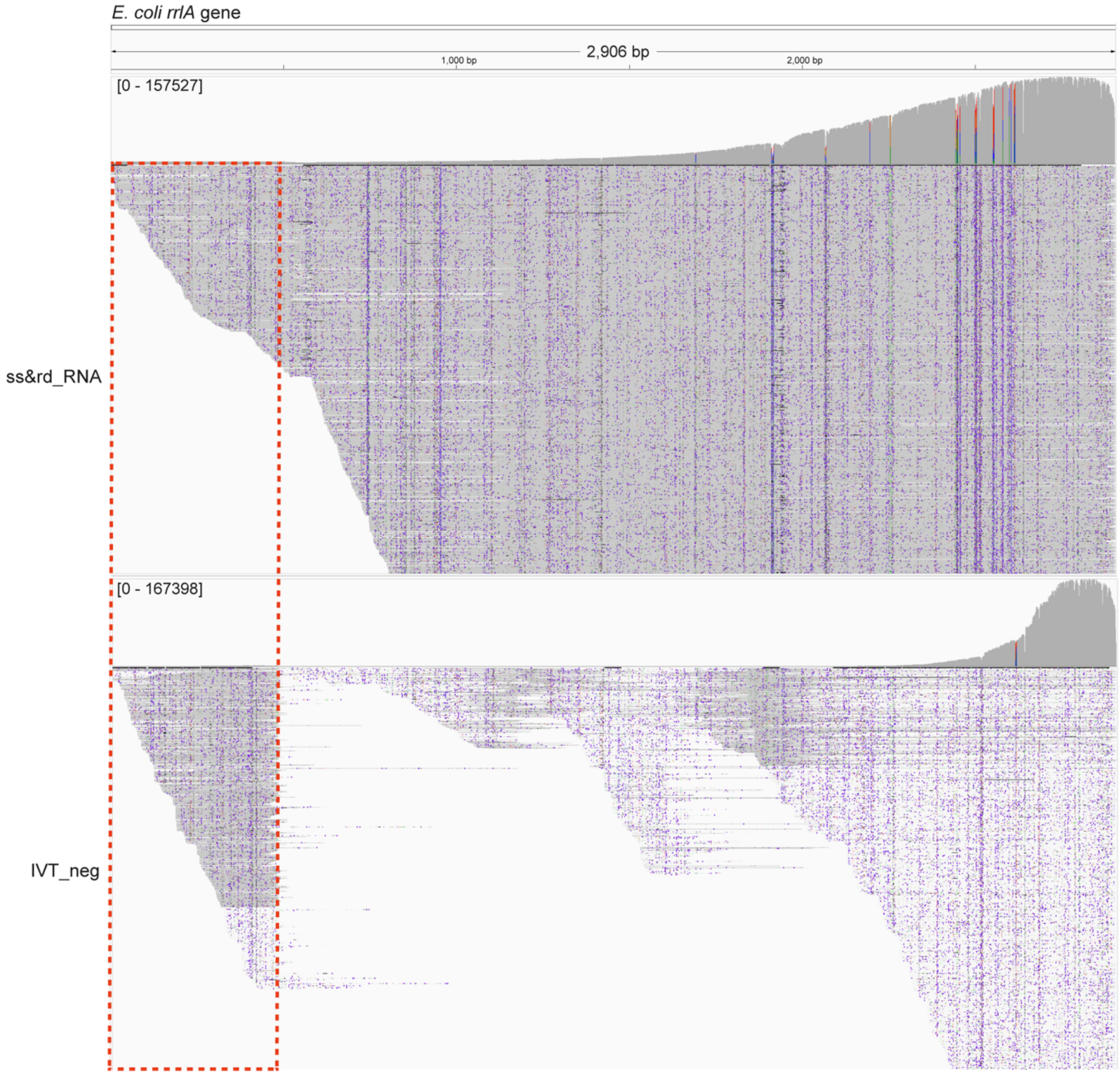

Figure S6. xPore issue exemplified by reads aligned to the *E. coli rrlA* gene. In this case, xPore outputted predictions in the region indicated by the dashed red box. Nonetheless, it failed to detect downstream sites and thereby missed the majority of the gene sequence. This issue has been described in the case of analyzing a pair of publicly available severe acute respiratory syndrome coronavirus 2 DRS data, which remains unsolved. The corresponding GitHub link is https://github.com/GoekeLab/xpore/issues/180.

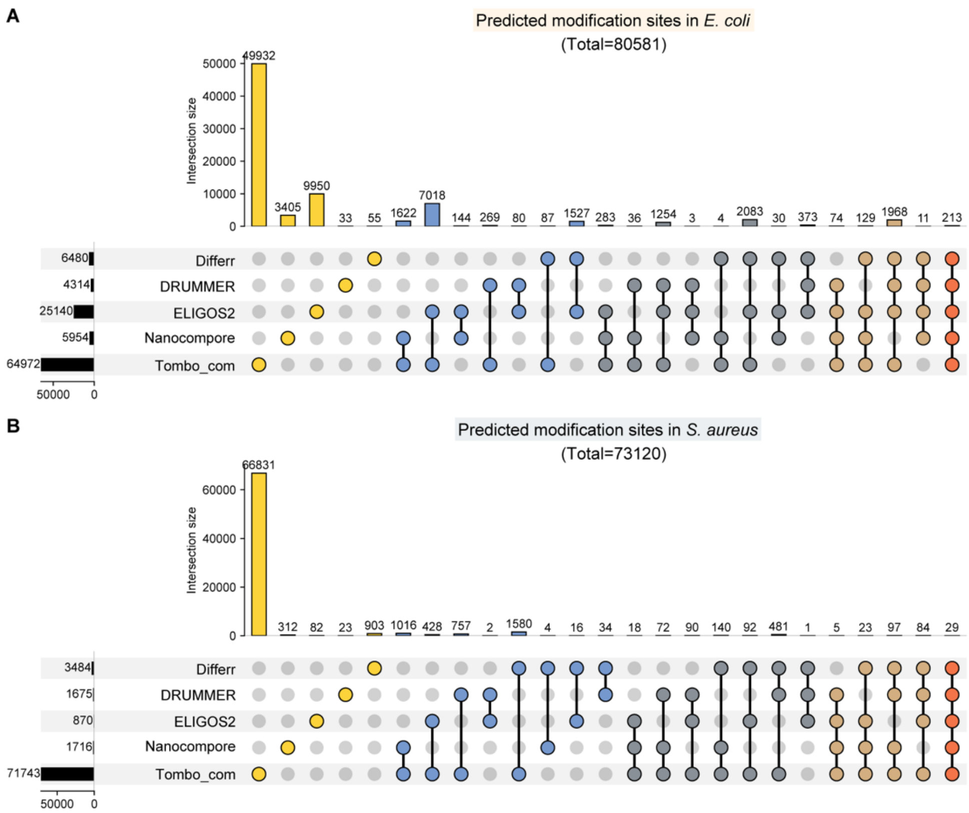

Figure S7. Intersections of modification sites detected by different computational tools. (A) and (B) UpSet plot of modification sites in the *E.coli* and *S. aureus* transcriptomes.

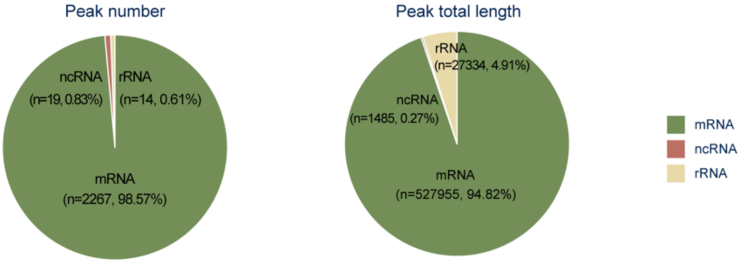

Figure S8. Methylated RNA Immunoprecipitation Sequencing (MeRIP-Seq) peak number and total length to annotation features.

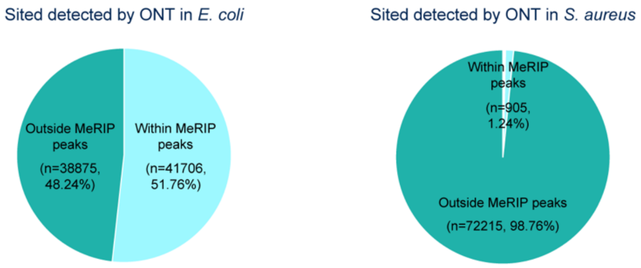

Figure S9. Ratios of ONT sites within and outside the MeRIP peaks. The predicted sites were supported by at least one computational tool.

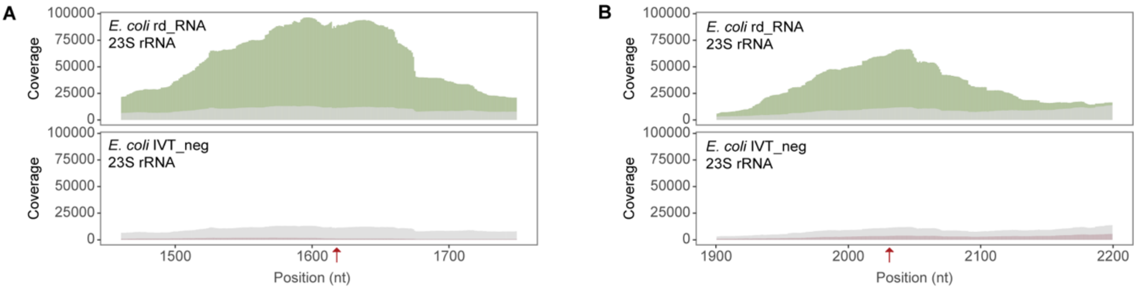

C

	Differr	DRUMMER	ELIGOS2	Nanocompore	Tombo_com
A1616	●	●	●		●
C1617	●		●		●
A1618					●
G1619					●
G1620					●

D

	Differr	DRUMMER	ELIGOS2	Nanocompore	Tombo_com
U2028	●	●	●		●
G2029	●	●	●		●
A2030	●	●	●		●
A2031					●
G2032					●

Figure S10. m6A-modified A1618 and A2030 on *E. coli* 23S rRNA detected by MeRIP-Seq and ONT-based computational tools. (A) and (B) MeRIP-Seq coverage plots of A1618 and A2030 and their adjacent regions. Red arrows indicate the locations of A1618 and A2030, respectively. (C) and (D) Prediction results of multiple computational tools at A1618 and A2030 and their adjacent positions.

Homer *de novo* Motif Results

Total target sequences = 75
Total background sequences = 4493
* - possible false positive

Rank	Motif	P-value	log P-pvalue	% of Targets	% of Background	STD(Bg STD)	Best Match/Details	Motif File
1	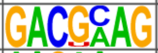	1e-19	-4.518e+01	16.00%	0.18%	1.4bp (1.0bp)	hsa-miR-3193 MIMAT0015077 Homo sapiens miR-3193 Targets (miRBase)(0.670) More Information | Similar Motifs Found	motif file (matrix)
2	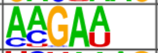	1e-14	-3.288e+01	32.00%	4.32%	1.7bp (1.9bp)	hsa-miR-2116 MIMAT0011160 Homo sapiens miR-2116 Targets (miRBase)(0.678) More Information | Similar Motifs Found	motif file (matrix)
3	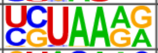	1e-12	-2.793e+01	12.00%	0.27%	1.1bp (1.1bp)	hsa-miR-1303 MIMAT0005891 Homo sapiens miR-1303 Targets (miRBase)(0.616) More Information | Similar Motifs Found	motif file (matrix)
4 *	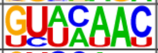	1e-8	-1.930e+01	10.67%	0.49%	1.2bp (1.4bp)	hsa-miR-3613-5p MIMAT0017990 Homo sapiens miR-3613-5p Targets (miRBase)(0.750) More Information | Similar Motifs Found	motif file (matrix)
5 *	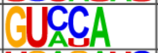	1e-6	-1.494e+01	10.67%	0.87%	2.1bp (2.0bp)	hsa-miR-4535 MIMAT0019075 Homo sapiens miR-4535 Targets (miRBase)(0.724) More Information | Similar Motifs Found	motif file (matrix)
6 *	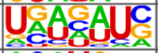	1e-4	-1.151e+01	17.33%	4.07%	1.3bp (1.5bp)	hsa-miR-4695-3p MIMAT0019789 Homo sapiens miR-4695-3p Targets (miRBase)(0.698) More Information | Similar Motifs Found	motif file (matrix)
7 *	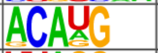	1e-4	-9.864e+00	12.00%	2.27%	1.3bp (1.9bp)	hsa-miR-4666-5p MIMAT0019741 Homo sapiens miR-4666-5p Targets (miRBase)(0.709) More Information | Similar Motifs Found	motif file (matrix)
8 *	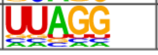	1e-1	-3.019e+00	1.33%	0.07%	0.0bp (0.0bp)	hsa-miR-18b* MIMAT0004751 Homo sapiens miR-18b* Targets (miRBase)(0.729) More Information | Similar Motifs Found	motif file (matrix)

Figure S11. Motifs identified at putative m6A sites on mRNAs.

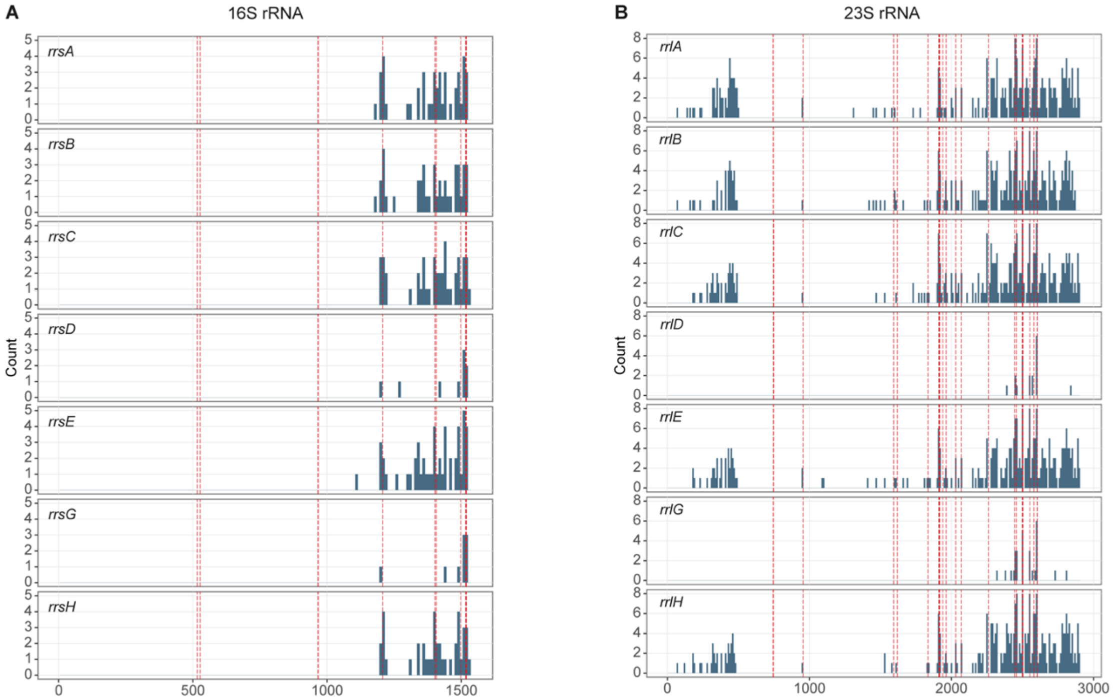

Figure S12. Profiling of potential modification sites on *E. coli* rRNAs. Modification sites supported by at least four computational tools were extracted from total predictions. (A) Potential modification sites on 16S rRNA. ONT reads were aligned to different gene copies of 16S rRNA. The full-length gene sequences are divided into 10-nt bins. The number of modification sites within each bin is shown on the Y-axis. The dark blue solid lines indicate the locations of inferred modifications by computational tools. The red dashed lines indicate the positions of known modifications. (B) Potential modification sites on 23S rRNA. ONT reads were aligned to different gene copies of 23S rRNA.

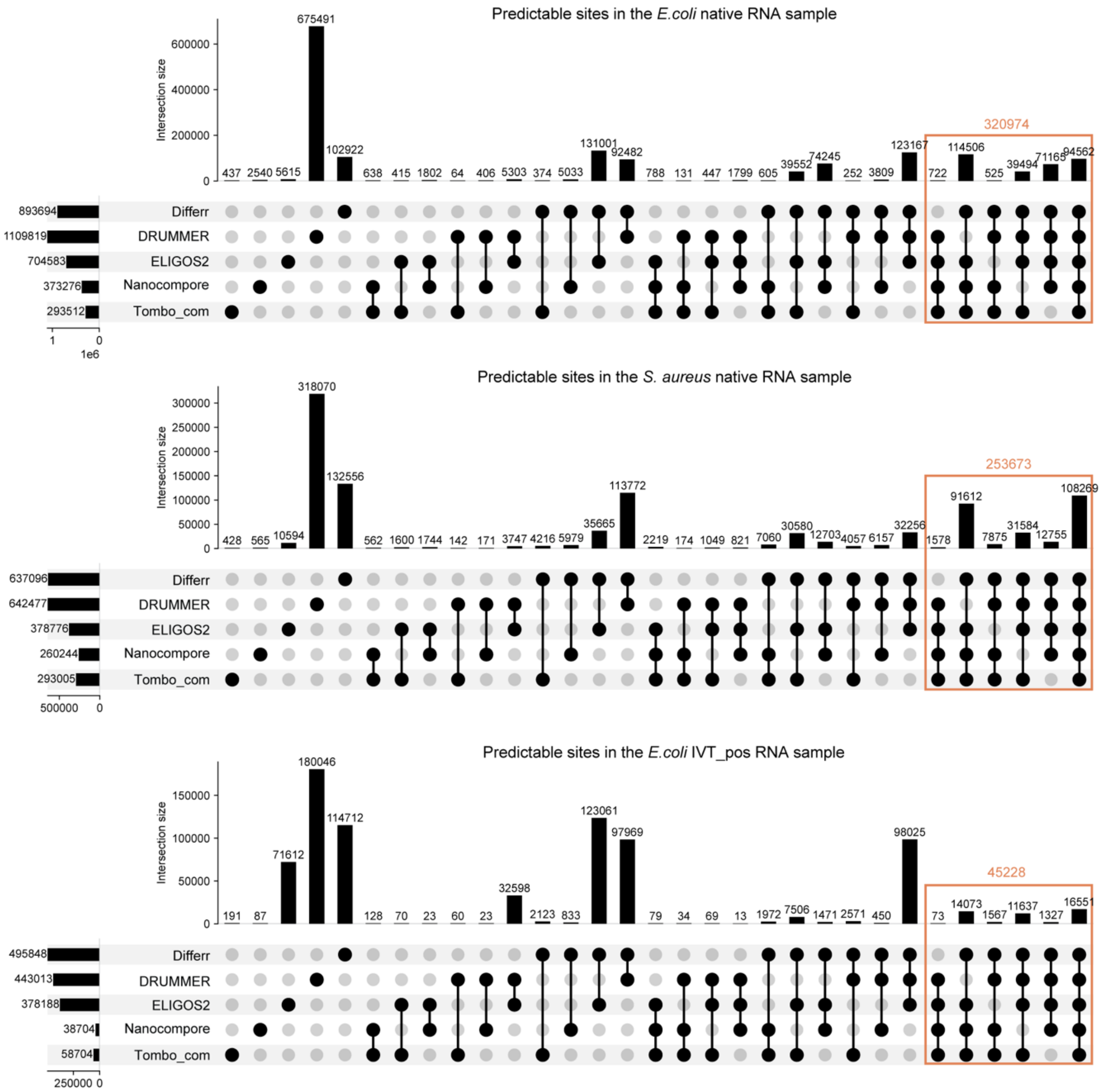

Figure S13. Intersections of predictable sites by individual computational tools. The orange rectangles highlight the sites detected by at least four tools, the sum of which is labeled above the rectangles.

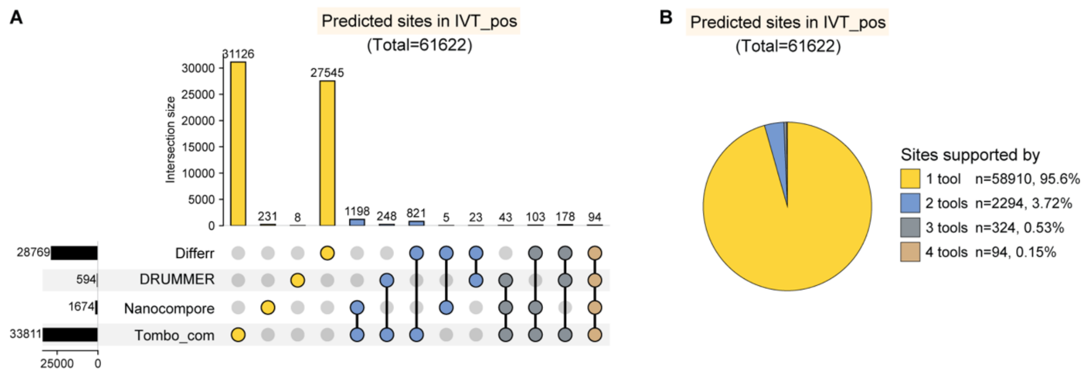

Figure S14. Intersections of modification sites predicted by different computational tools in the *E. coli* IVT_pos sample. (A) UpSet plot of modification sites. (B) Percentage of modification sites supported by multiple tools.

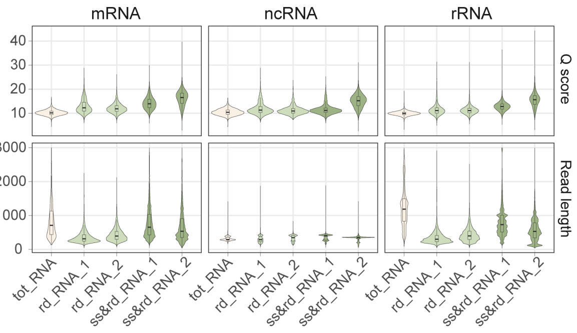

Figure S15. Read quality and length of *E. coli* DRS reads aligned to mRNA, ncRNA, and rRNA, respectively.

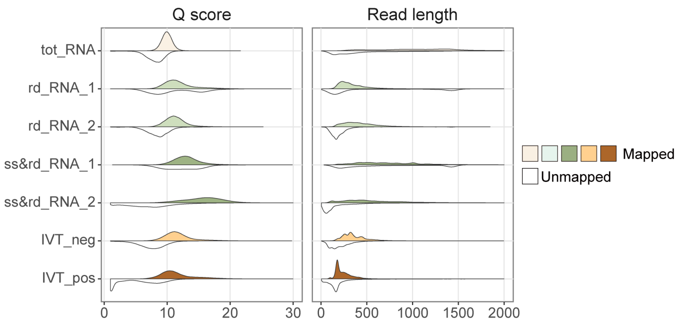

Figure S16. Read quality and length of *E. coli* DRS mapped and unmapped reads.

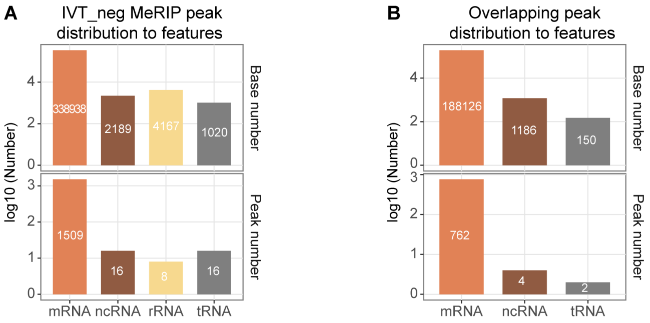

Figure S17. False-positive peak calling of the IVT_neg sample. (A) IVT_neg MeRIP peak distribution to features. (B) Distribution of overlapping peaks between IVT_neg and native samples to features.

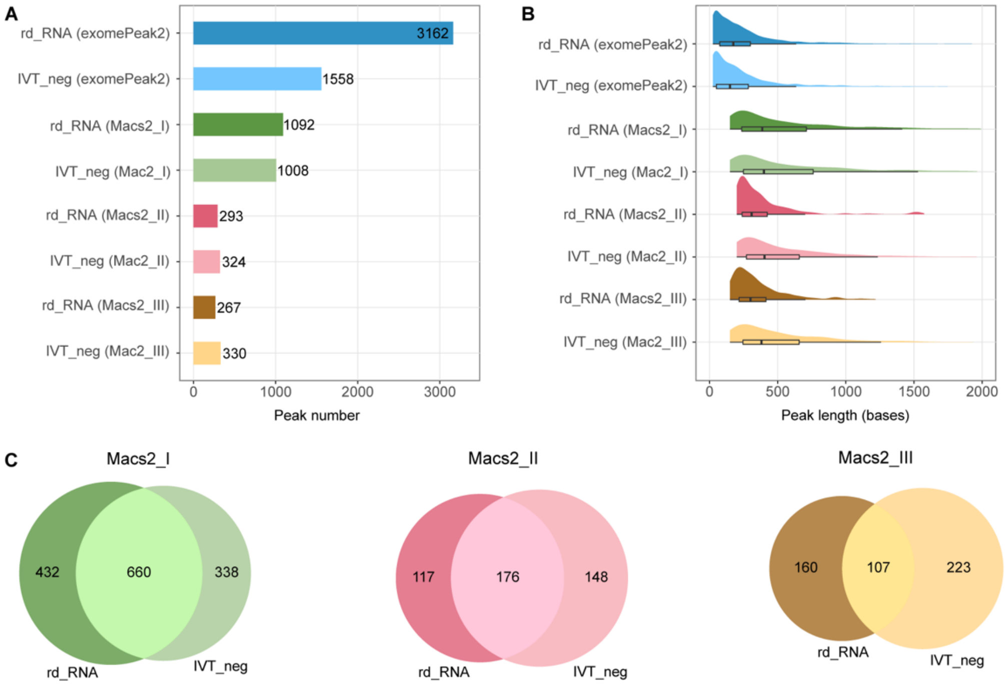

Figure S18. *E. coli* MeRIP-Seq peak calling results obtained with different analytic tools and parameter settings. The MeRIP-Seq data was processed using exomePeak2 v1.9.1 or Macs2 v2.2.7.1. The parameters “p_cutoff=0.00001, log2FC_cutoff=1, fragment_length=150” were used for exomePeak2. Three different parameter settings were applied to Macs2: “-B --SPMR -f BAM --nomodel --nolambda --extsize 150 -q 0.01” for Macs2_I; “-B --SPMR --keep-dup all -f BAM -p 1e-5 --nomodel” for Macs2_II (15); “--keep-dup all -f BAM --nomodel --extsize 150 -q 0.05 --fe-cutoff 2” for Macs2_III (21). (A) m6A peak numbers of differentially processed datasets. (B) m6A peak lengths of differentially processed datasets. (C) Venn diagrams showing the m6A peaks in native and modification-free RNA libraries obtained with different Macs2 parameter settings.

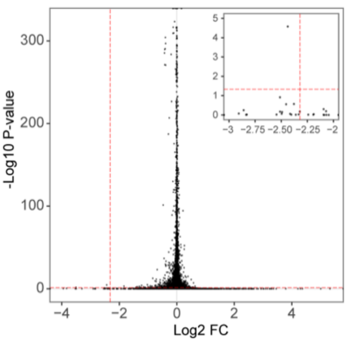

Figure S19. Analytic result of m6A-sensitive RNA-Endoribonuclease-Facilitated sequencing (m6A-REF-seq) in *E. coli*. The experimental design and data analysis pipeline were referenced from (21). A modification-free IVT RNA sample was processed and analyzed in parallel with the native sample to eliminate false positives. Red dashed lines indicate the cutoffs for high-confidence m6A sites.
